# Thermal infrared directs host-seeking behaviour in *Aedes aegypti* mosquitoes

**DOI:** 10.1038/s41586-024-07848-5

**Published:** 2024-08-21

**Authors:** Avinash Chandel, Nicolas A. DeBeaubien, Anindya Ganguly, Geoff T. Meyerhof, Andreas A. Krumholz, Jiangqu Liu, Vincent L. Salgado, Craig Montell

**Affiliations:** 1 Department of Molecular, Cellular, and Developmental Biology, University of California, Santa Barbara, CA USA; 2 Neuroscience Research Institute, University of California, Santa Barbara, CA USA; 3 BASF, Research Triangle Park, NC USA; 4 Present Address: Südzucker, Mannheim, Germany; 5 Present Address: Department of Biology, Duke University, Durham, NC USA

**Keywords:** Ion channels in the nervous system, Behavioural genetics

## Abstract

Mosquito-borne diseases affect hundreds of millions of people annually and disproportionately impact the developing world^1,2^. One mosquito species, *Aedes* *aegypti*, is a primary vector of viruses that cause dengue, yellow fever and Zika. The attraction of *Ae. aegypti* female mosquitos to humans requires integrating multiple cues, including CO_2_ from breath, organic odours from skin and visual cues, all sensed at mid and long ranges, and other cues sensed at very close range^3–6^. Here we identify a cue that *Ae. aegypti* use as part of their sensory arsenal to find humans. We demonstrate that *Ae. aegypti* sense the infrared (IR) radiation emanating from their targets and use this information in combination with other cues for highly effective mid-range navigation. Detection of thermal IR requires the heat-activated channel TRPA1, which is expressed in neurons at the tip of the antenna. Two opsins are co-expressed with TRPA1 in these neurons and promote the detection of lower IR intensities. We propose that radiant energy causes local heating at the end of the antenna, thereby activating temperature-sensitive receptors in thermosensory neurons. The realization that thermal IR radiation is an outstanding mid-range directional cue expands our understanding as to how mosquitoes are exquisitely effective in locating hosts.

## Abstract (web summary)

The mosquito *Aedes aegypti* can detect humans through infrared radiation for highly effective mid-range navigation.

## Main

**Fig. 1:**
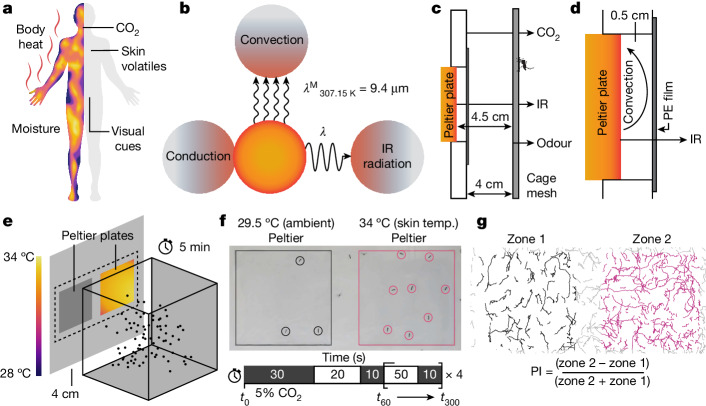
Set-up for testing IR radiation as a potential host-associated cue. **a**, Known host-associated sensory cues. **b**, Modes of thermal energy transfer: convection, conduction and IR radiation. The peak emission wavelength (*λ*^M^) of emitting bodies at 34 °C is around 9.4 µm. **c**, The host-associated cues presented during the assay: human odour, 5% (v/v) CO_2_ and heat in the form of IR radiation. Assay cages were 4 cm from the arena wall that housed the Peltier device to mitigate the effect of convective cues. Human odour was applied uniformly on the outside of the mesh of the assay cage from a used nitrile glove. CO_2_ was delivered through perforated tubing, which formed a perimeter around both the control and IR zones. **d**, The Peltier device housing. An IR-transparent polyethylene (PE) film blocked convective cues from reaching the mosquitoes. **e**, Schematic of the behavioural assay. Mosquitoes were presented with IR and their host-seeking behaviour was video recorded for 5 min. **f**, Representative video frame taken from an experiment in which females were exposed to human odour and 5% CO_2_. One zone was exposed to 34 °C radiant heat from a Peltier device. The Peltier device behind the other zone was off, and equilibrated to the ambient temperature (temp.) (29.5 °C). The position of each host-seeking mosquito was recorded during the experimental window. In all of the experiments in which CO_2_ was provided, it was applied using the indicated time series (in seconds) unless otherwise stated. **g**, The PI, calculated from the indicated formula, using the total number of host-seeking observations in each zone during the 5 min experiment. PI < 0 indicates preference for zone 1; PI > 0 indicates preference for zone 2.

*Aedes* *aegypti* is an invasive mosquito species that transmits flaviviruses, impacting a growing proportion of the world’s population^1,2^. As female mosquitoes blood feed multiple times, they often shuttle viruses causing diseases ranging from dengue to yellow fever, Zika and chikungunya^2^. *Ae. aegypti* integrate multiple sensory cues to locate and navigate towards humans^3–6^ (Fig. 1a). Integration is essential as any single stimulus is inadequate to differentiate humans from other targets. Detection of exhaled CO_2_ elevates their locomotor activity and increases their responsiveness to other host-derived stimuli, such as visual cues^3–6^. However, *Ae. aegypti* has poor visual acuity, limiting its usefulness in discriminating between people and other hosts^7^. Organic olfactory cues are particularly important for finding humans. However, the efficacy of CO_2_ and olfactory cues in providing directional information is limited by air-current disturbances that exceed the mosquito’s flight speed, or if the host is moving quickly^8^. When mosquitoes are very close to the skin surface, they detect moisture and convective body heat^4,9^.

Thermal energy is transferred through conduction, convection and radiation (Fig. 1b). Mosquito attraction to heat depends at least in part on the antenna^10,11^. The terminal antennal segment contains neurons that respond to cooling and warming^12–15^. Conduction requires contact and is not useful during host-seeking flight. Convective currents move upwards and are sensed only at distances of less than 10 cm (ref. ^16^). Detecting convective heat from hosts at short range is promoted by a general attraction to warmth, and by avoiding both cool and very hot temperatures^17^. Elimination of either of two ionotropic receptors from cooling-responsive neurons impairs attraction to convection heat^14,18^. Moreover, disruption of the *Aedes* TRPA1 channel impairs avoidance of very hot temperatures at close range (50−60 °C), but does not impair the responses to surfaces ≤45 °C (ref. ^17^). The neurons that depend on TRPA1 for avoiding noxious heat remain to be identified. In *Anopheles gambiae*, *trpA1* is expressed at the antennal tip^15^, although its thermosensory role is unclear.

Conductive heat requires contact, and convective heat is sensed at close range^16^. Thus, if mosquitoes sense thermal IR radiation, then surface body temperature could be detected at greater distances, as radiant heat is not limited by the physical constraints of convection and conduction. Thermal IR emitted by humans (~34 °C skin surface) has a peak emission wavelength of around 9.4 μm, with 90% of its energy between 3–30 μm (ref. ^19^). This electromagnetic radiation is much lower energy than the ~300–700 nm wavelengths that activate rhodopsins^20^. Thus, if mosquitoes detect thermal IR, this sensation should not rely on phototransduction.

Few animals are thought to sense IR for navigation or sensing prey. These include rattlesnakes^21,22^, certain beetles^23,24^, western conifer seed bugs^25^, kissing bugs and ticks^26,27^. By contrast, it has been reported that mosquitoes, including *Ae. aegypti*, are not attracted to IR^9,28,29^. However, in these studies, thermal IR was presented alone rather than in the presence of other host-associated stimuli^9,28,29^. Given the importance of multisensory integration in host seeking^3–6^, we wondered whether mosquitoes might exhibit attraction to thermal IR, but only in combination with other host cues.

## IR is integrated with other host cues

**Extended Data Fig. 1:**
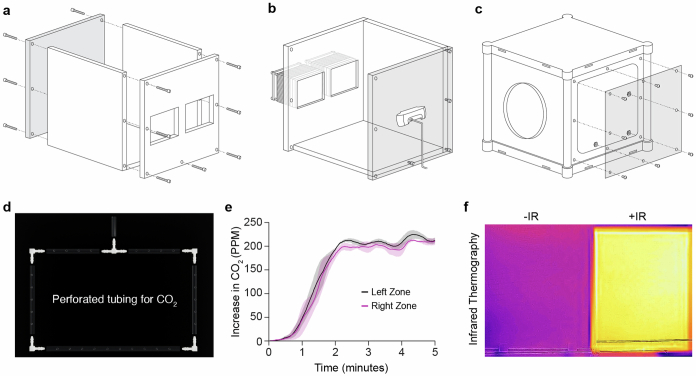
IR-based behavioural assay setup. **a**, Design and construction of IR assay arena. **b**, Diagram of Peltier plates and recording camera position in the IR assay arena (image shown with one side wall removed for clarity). **c**, Schematic of assay cage used in IR behaviour experiments. The clear acrylic panel is shown in grey. **d**, Perforated tubing surrounding the two Peltier plates. **e**, Concentrations of CO_2_ recorded from a CO_2_ sensor placed inside an assay cage and exposed to the 5-minute CO_2_ paradigm used in the study. There were no differences in CO_2_ concentration in the left and right zones. Means ± SEMs (n = 3 independent measurements). **f**, Infrared thermograph of the thermal output of each zone of the IR source used in the distance setup. The right zone was held at ~34 °C, while the left zone was allowed to equilibrate with the ambient incubator conditions. Image acquisition and temperature estimation were made using a FLIR One infrared smartphone camera. Source Data

To determine whether *Ae. aegypti* can detect IR while host seeking, we developed a behavioural assay to present thermal IR to the mosquitoes in the context of other host-associated stimuli. The assay set-up comprised two components. The first was a custom-fabricated arena in which we mounted two temperature-controlled plates (Peltier devices; Extended Data Fig. 1a,b). Second, we made specialized assay cages by replacing the mesh panel on one side of a mosquito-rearing cage with clear acrylic to enable unobstructed video recording (Extended Data Fig. 1c). We performed assays by lowering the cage into the arena and video recording mosquito movement. One Peltier device was typically set at 34 °C (approximate skin temperature) and provided the source of thermal IR. We used 34 °C rather than a cool IR source as the wavelength of IR is a function of the temperature of the source. Thus, only temperatures at around 34 °C would radiate IR with a peak (9.4 μm) and spectrum (3 μm to 30 μm) equivalent to that generated by the surface temperature of humans. The second Peltier device was not heated and was equilibrated to an ambient temperature of 29.5 °C, as it is very close to the optimal environmental temperature for *Ae. aegypti* (29.2 °C)^30^.

**Extended Data Fig. 2:**
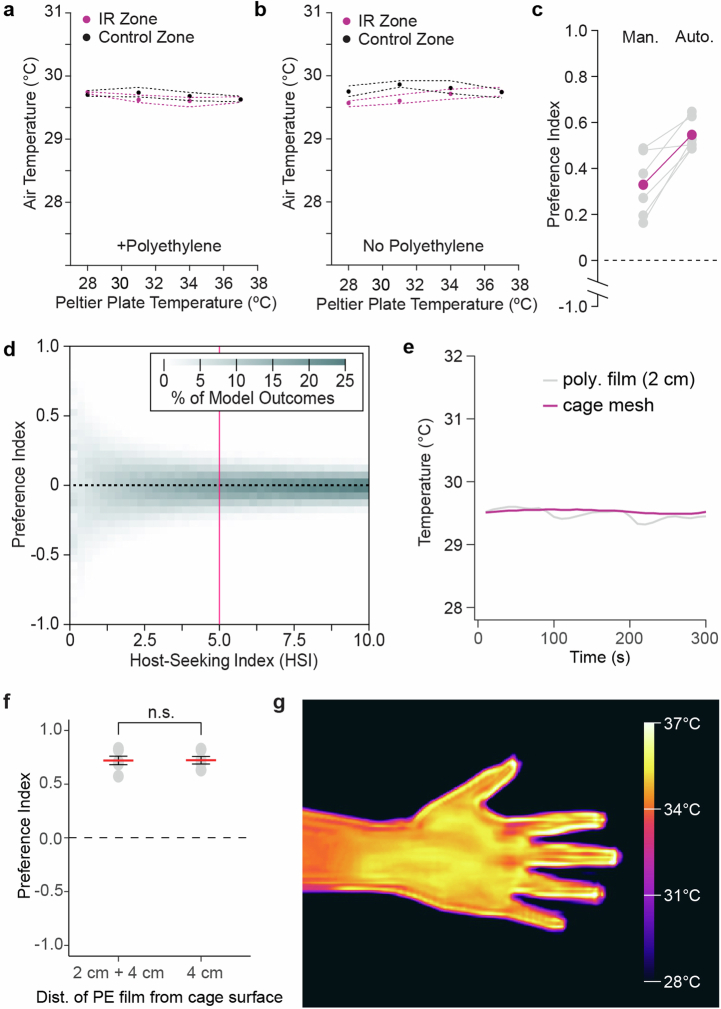
Temperatures of the behavioural setup and of a human hand and arm, and a heatmap of model outcomes under various host-seeking activities. **a**, Air temperature recordings taken from a distance of 4 cm from the arena wall that housed the two Peltier plates used in the behavioural assay. A thin polyethylene film was placed 0.5 cm in front of the Peltier plates. The circles indicate the mean recorded air temperatures over a 5-minute recording period. The dashed lines indicate the maximum and minimum recorded temperatures during a 5-minute window. **b**, Same analysis as in panel ‘a’ without the polyethylene film. **c**, Comparison showing the sensitivity of the manual (Man.) versus the automated scoring method (Auto.) for the same experiments, n = 6 biological replicates. **d**, The input number of fictive mosquitoes used in the simulation ranged from 1 to 30. At each input number, the model was iterated 10,000 times. Simulation data were then analysed for the PI and the HSI. The interpretation of these data helped shape the minimum HSI used in this study (HSI = 5, red line). **e**, Temperature recordings over a 5-minute period at the cage mesh (red) and at the second polyethylene film (turquoise), which is placed 2 cm from the cage mesh. **f**, IR-preference assay performed with the first and second polyethylene films (2 cm and 4 cm from the cage mesh) and with just the first polyethylene film (4 cm from the cage mesh). Means ± SEMs (n = 6 biological replicates) Two-tailed Student’s t-test, n.s., not significant. **g**, IR thermography of a human hand and arm demonstrating non-homogenous skin temperatures. Exact P-values provided in Source Data. Source Data

We devised our behavioural assay to transmit only thermal IR to the mosquito cage from the heated Peltier plate. To prevent thermal conduction, we placed the mosquito cage 4 cm away from the arena wall that housed the Peltier plates (Fig. 1c). To block convection from the warm surface from reaching the mosquitoes we positioned the Peltier plates an additional ~0.5 cm away from the interior surface of the arena, and covered the opening of the recesses with thin polyethylene (PE) film (Fig. 1d). We chose PE because it has a high (nearly 100%) transmission rate for thermal IR between 3 and 30 μm (ref. ^31^), which is emitted by a 34 °C source. Moreover, its mass is so low that it does not transfer a measurable amount of heat to the air between it and the mesh, so it effectively blocks convection. To determine the efficacy of this convective barrier, we recorded the air temperature 4 cm from the arena’s wall using a temperature probe positioned orthogonally to the Peltier surface. We found no difference in air temperature between the reference zone (29.5 °C) and the other zone when the Peltier temperature was 28–37 °C (Extended Data Fig. 2a). Even without the PE film, the temperatures at both zones were unchanged (Extended Data Fig. 2b). Thus, the 4.5 cm distance of the Peltier surface from the mosquito cage was sufficient to negate convective effects, demonstrating that the convective barrier was a redundant convection control.

To conduct the behavioural assays, we transferred 80 female mosquitoes into the assay cages and allowed them to acclimatize for ≥24 h, while they were allowed to feed on 10% sucrose ad libitum. We then placed the cage inside the arena and monitored host-seeking behaviour for 5 min by recording mosquito movement after landing on the cage’s mesh directly opposite the Peltier devices (Fig. 1e,f). We defined host-seeking behaviour as a mosquito landing, walking and extending its proboscis through the mesh of the cage (Supplementary Video 1), which is reminiscent of a female landing on a human and then walking while sampling the skin surface with its labellum. We did not score instances when mosquitoes were present in either zone but were stationary.

We found that the instantaneous rate of host seeking and the distribution of mosquitoes varied greatly throughout the 5 min experimental window. As such, manual measurements at set intervals less accurately capture broader trends in preference and activity (Extended Data Fig. 2c). To circumvent this, we developed a custom video-tracking program to capture all movement data throughout the experiment. Using this tool, we recorded the position of each mosquito that was actively host seeking on the cage mesh throughout the 5 min experimental window (Fig. 1g; details of the tracking methods and scoring parameters are provided in the ‘Development of object-tracking MATLAB scripts’ section of the Methods). We used the preference index (PI) and host-seeking index (HSI) to characterize experimental outcomes and defined the PI as the difference in total host-seeking observations between the two zones, normalized to the total number of observations (Fig. 1g). The HSI represents the average number of mosquitoes actively host seeking at any given time (the calculation is shown in the Methods). For all experiments concerning preference, we defined a minimum HSI of 5 as the threshold for inclusion in this study (Extended Data Fig. 2d; see the ‘Optimization of scoring parameters’ section of the Methods).

**Fig. 2:**
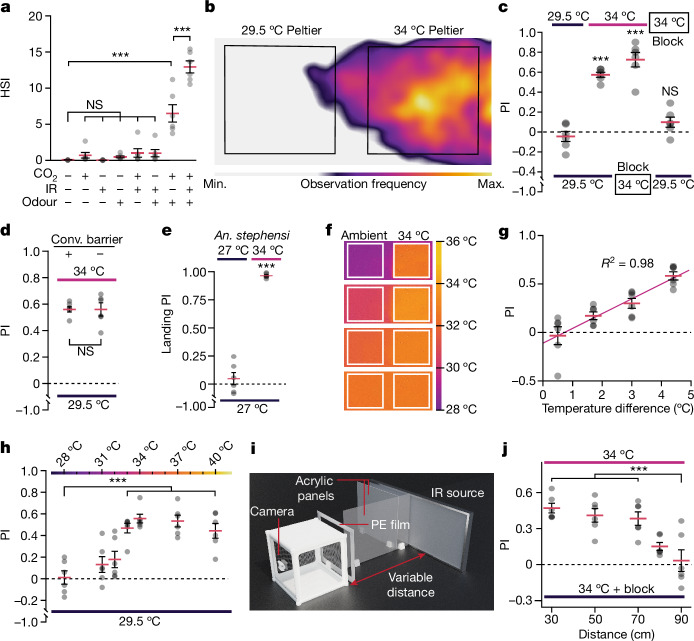
Integrating IR with other cues to direct host-seeking behaviour. **a**, The effects of host-associated cues on host-seeking: 5% CO_2_, human odour and a 34 °C IR source. The HSI represents the average number of female mosquitos host seeking during 5 min experiment. **b**–**d**,**f**–**h**, Experiments in which both zones were exposed to CO_2_ and human odour, and one zone was exposed to IR at the indicated temperatures. Ambient temperature, 29.5 °C. **b**, The host-seeking frequency during an assay. **c**, There was no preference when both sides were at 29.5 °C. There was a strong preference for the 34 °C IR zone over the 29.5 °C zone. Obstructing IR from one of the two 34 °C IR sources produced a preference for the unobstructed source. Blocking IR from the 34 °C zone abolished the preference over the 29.5 °C zone. **d**, Removing the convective (conv.) barrier (−) did not increase the preference for the 34 °C zone. **e**, Female *An. stephensi* were exposed to two zones with human odour and 5% CO_2_. The zones were at 27 °C ambient temperature or exposed to 34 °C IR. **f**, IR thermographs under different ambient temperatures (left zone). The right zone was held at 34 °C. **g**, Assays performed under different ambient temperatures. Linear regression between the PI and temperature differences between the two zones. **h**, Assays were performed using different IR source temperatures (28–40 °C) in a 29.5 °C environment. **i**, The set-up for determining the distances at which *Ae. aegypti* detect 34 °C IR. Each side of the cage was exposed to human odour and CO_2_. IR was blocked on one side with an acrylic panel. Distances of 30–90 cm were used. **j**, The distances at which *Aedes* detect IR. Mosquitoes exposed to CO_2_, human odour and 34 °C IR in one zone, and 29.5 °C in the other zone. Data are mean ± s.e.m. Statistical analysis was performed using one-way analysis of variance (ANOVA) with Tukey’s multiple-comparison test (**a**, **c**, **h** and **j**) and parametric two-tailed Student’s *t*-tests (**d**,**e**); NS, not significant; ***P < 0.001. For **a**–**e**, **g**, **h** and **j**, *n* = 6 biological replicates for each group. Exact *P* values are provided as source data. Source Data

To empirically determine which combinations of host-associated cues evoked the most robust HSIs, we tested each permutation of 5% CO_2_ (approximate concentration of exhaled breath)^32^, human odour from a worn glove and IR from a 34 °C Peltier source. Unsurprisingly, in the absence of any host-associated cue, the HSI was approximately 0% (0.04 ± 0.01%; Fig. 2a). Any single cue alone (CO_2_, IR and human odour) evoked either no HSI or a very weak response (Fig. 2a). When we presented the mosquitoes with CO_2_ + IR or human odour + IR, these conditions also elicited only weak HSIs (Fig. 2a). Human odour and CO_2_ together evoked a significant increase in HSI (Fig. 2a; HSI = 6.50 ± 1.20%). Importantly, we observed a highly significant, twofold increase in the HSI when we added IR to the CO_2_ and human odour combination (Fig. 2a; HSI = 12.93 ± 0.83%).

**Extended Data Fig. 3:**
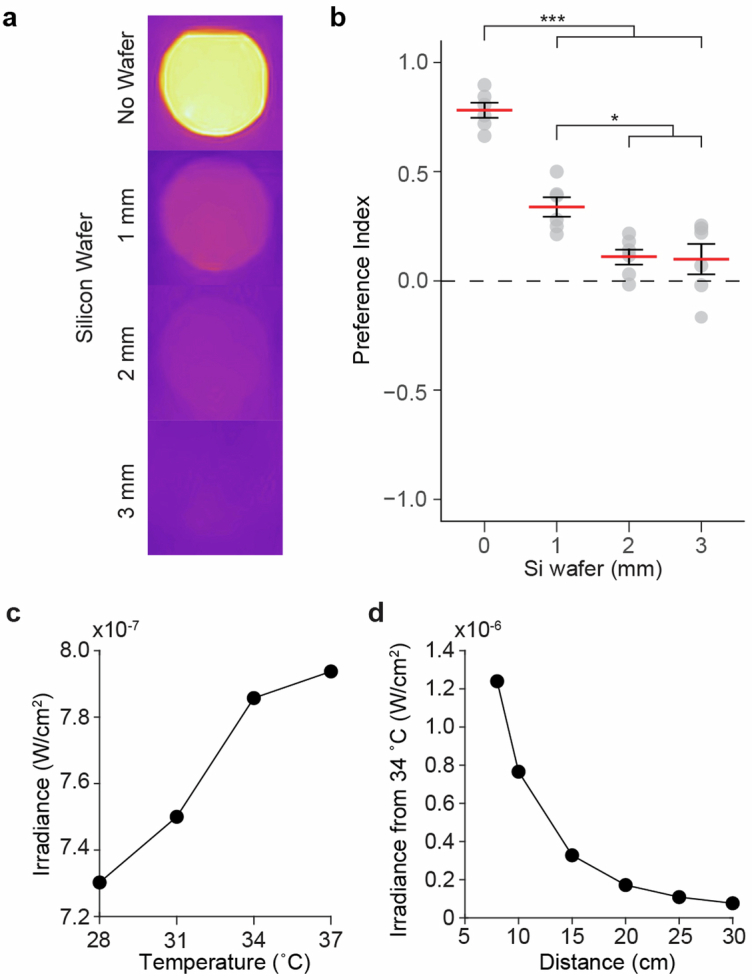
Effects on behaviour of dosedependent block of IR preference, and effects of temperature and distance on IR intensity. **a**, IR thermograph of the IR source used in the IR block setup. The IR zone was held at 34 °C and covered using different thickness of IR blocking windows. Acquisition of the images was made using a FLIR One infrared smartphone camera and the background was normalized using FLIR ignite software. **b**, IR preference assay performed using different thickness of IR blocking Si wafers covering the 34 °C IR source. Means ± SEMs (n = 6 biological replicates). One-way ANOVA with Tukey’s HSD test. *P < 0.05 and ***P < 0.001. **c**, IR intensity measurements with a pyroelectric sensor when the temperature of the IR source varies between 28°– 37 °C. **d**, IR intensity measurements using a pyroelectric sensor when the distance of the IR source at 34 °C is 8 cm to 30 cm from the sensor. Exact P-values provided in Source Data. Source Data

Using these stimuli in combination, we next sought to determine whether mosquitoes prefer IR while host seeking. When we provided an IR cue on one side (Peltier at 34 °C), there was a significant shift in preference for that zone (Fig. 2b,c; PI = 0.57 ± 0.02; Supplementary Video 2). There was no preference for either zone 1 or 2 when both plates were turned off and were at the 29.5 °C ambient temperature (Fig. 2c; PI = −0.05 ± 0.05). There was therefore no bias for either the left or right zones under neutral conditions. Obstructing the IR emitted from the 34 °C zone with a combination of materials with low IR transmission (acrylic and aluminium foil) eliminated the strong preference (Fig. 2c; PI = 0.08 ± 0.05). When we blocked most of the thermal IR from the 34 °C with 2-mm- and 3-mm-thick silicon wafers (Extended Data Fig. 3a), the PI for the IR side was substantially reduced (Extended Data Fig. 3b; no wafer, PI = 0.78 ± 0.03; 2 mm wafer, PI = 0.11 ± 0.03; 3 mm wafer, PI = 0.09 ± 0.06). A smaller reduction in the PI occurred when we used a 1 mm silicon wafer, which is less effective in blocking IR (Extended Data Fig. 3a,b; PI = 0.33 ± 0.04). When we exposed both zones to 34 °C radiant heat and blocked the IR on one side, there was a strong preference for the unblocked side (Fig. 2c; PI = 0.72 ± 0.07). All of these data demonstrate that female *Aedes* detect thermal IR while navigating during host seeking.

Throughout this study, we used CO_2_, odour and IR (34 °C) in all of the behavioural experiments unless otherwise indicated. Moreover, we performed all further experiments with the convective barrier in place, even though, when we removed the convection barrier, mosquito preference was unchanged (Fig. 2d), and the temperature at the surface of the cage closest to the Peltier did not increase above the ambient temperature (Extended Data Fig. 2b). Nevertheless, to provide an additional test that convection is not influencing the behavioural response to the 34 °C source of thermal IR, we inserted a second PE film positioned 2 cm from the first PE layer and 2 cm in front of the cage mesh where the mosquitoes land. We recorded the temperature at the surface of the second mesh and at the cage surface over a 5 min time span. We found that the temperature of the PE film 2 cm in front of the cage was not higher than the surface of the cage mesh, demonstrating that the PE film was not a source of convection heat (Extended Data Fig. 2e). Moreover, the PI exhibited by females for the zone with IR (plus CO_2_ and human odour) over the zone without IR (CO_2_ and human odour only) was the same regardless of whether there was a second PE film 2 cm from the cage surface, or if there was only one PE film 4 cm from the cage surface (Extended Data Fig. 2f). These data further demonstrate that the preference for one zone was due to thermal IR and not convection heat emanating from the PE film.

The automatically determined PI includes only walking mosquitoes, which are typically extending their proboscis through the mesh, reminiscent of blood-seeking behaviour. This is unlike previously used manual counting approaches that did not discriminate between random landings from mosquitoes that display behaviour associated with blood seeking. When non-host seeking *Aedes* females land, they sometimes remain in the same location for many minutes unless otherwise disturbed. We compared the sensitivity of this automated assay system with manually quantifying the number of mosquitoes in each zone every 30 s from a set of videos where 34 °C IR was presented along with human odour and CO_2_. We found that manual counting demonstrated a strong bias for the IR side, but under-represented the preference for the IR zone compared with our automated scoring method (Extended Data Fig. 2c).

*Ae. aegypti* is most active near dawn and dusk, whereas *Anopheles* *stephensi*, which is one of the major vectors for the malaria-causing parasite, is most active at night. To address whether this night-time biting mosquito also uses IR as a host-seeking cue, we modified our conditions (Methods) and performed IR-preference experiments at an ambient temperature of 27 °C, as we found that these mosquitoes were more active at this temperature than at 29.5 °C. We exposed one side to IR from a 34 °C source, and both sides to human odour and 5% CO_2_. We found that *An. stephensi* displayed a very strong bias for landing on the IR zone (Fig. 2e; PI = 0.96 ± 0.01). However, in contrast to *Ae. aegypti*, after landing on the IR zone, *An. stephensi* rarely walked around probing, and would quickly resume their flight. Thus, while *An. stephensi* displayed an extremely strong preference for landing on the IR zone, they did not exhibit the same type of host-seeking behaviour that we characterized for *Ae. aegypti*. Furthermore, the overall host-seeking response of *Anopheles* was much lower than *Aedes*. For the remainder of this study, we focused on *Ae*. *aegypti*.

## IR source at skin temperature is most attractive

*Ae. aegypti* show strong activity around dawn and dusk when ambient temperatures are lower than the midday highs. To determine how environmental temperature affects *Ae. aegypti* IR sensitivity, we studied their behaviour across various ambient temperatures by adjusting the temperature of the incubator housing the behavioural assay set-up. We then measured the difference in temperature between the two zones using IR thermography (Fig. 2f). These images represent the IR landscape that mosquitoes navigate during host seeking. We also quantified the levels of IR generated by the Peltier device at various temperatures (28 °C to 37 °C), using a pyroelectric IR sensor with a spectral range spanning 1 to 25 μm. Our findings reveal a nearly linear increase in IR intensity as the temperature of the Peltier device increased from 28 °C to 37 °C (Extended Data Fig. 3c). We also determined the IR signal intensity at different distances (8 cm to 30 cm) from the 10 cm × 10 cm Peltier device set at 34 °C, which we used for our experiments. The IR intensity declined with the inverse square of the distance (Extended Data Fig. 3d).

When the ambient environment temperature was lower than the IR target (29.5 °C versus 34 °C, respectively), the attraction of the mosquitoes to the IR target was high (Fig. 2g; temperature difference, 4.4 °C; PI = 0.58 ± 0.04). Conversely, when the ambient environment approximated the temperature of the IR source (~34 °C), the preference for the zone with IR was lost (Fig. 2g; temperature difference, 0.05 °C; PI = −0.03 ± 0.09). Over the range of temperatures studied, the PI was directly proportional to the measured temperature difference (Fig. 2g; *R*^2^ = 0.98). Thus, the usefulness of IR sensation during host seeking is greatest when the thermal target is considerably warmer than the ambient environment.

*Ae. aegypti* females prefer to blood feed on humans over other animals^33^. However, if humans are unavailable, they target other homeothermic animals, especially mammals^34^. Human skin temperature is slightly cooler than core body temperatures (~34 °C and ~37 °C, respectively), and varies across the body’s exterior, such as the hand and arm (Extended Data Fig. 2g). The surface temperature of different mammalian species in the same environment can differ from humans by as much as 10 °C (ref. ^35^). This raises the possibility that *Ae. aegypti* females might associate a discrete range of thermal IR with preferred hosts.

To determine the temperatures that are most attractive to female *Aedes* during host seeking, we measured the PI values across a range of target temperatures contrasted with a consistent ambient environment (29.5 °C). There were slight increases in the preferences for 31 °C and 32 °C that were not statistically significant (Fig. 2h; 31 °C, PI = 0.13 ± 0.07; 32 °C, PI = 0.18 ± 0.08). When we raised the Peltier only 1 °C higher to 33 °C, the mosquitoes exhibited a large shift in their preference for this thermal IR (Fig. 2h; PI = 0.45 ± 0.04). The mosquitoes demonstrated the highest preference for a 34 °C source of thermal IR (Fig. 2h; PI = 0.56 ± 0.04). The preference for thermal IR trended slightly downward at higher IR intensities (Fig. 2h; 37 °C, PI = 0.53 ± 0.05; 40 °C PI = 0.44 ± 0.07). This result is notable as 37 °C and 40 °C have temperature differences from the ambient temperature of 29.5 °C (7.5 °C and 10.5 °C, respectively) that are greater than the difference between 34 °C and 29.5 °C (4.5 °C). Thus, the degree of mosquito attraction to a thermal IR source is not simply proportional to the temperature difference or contrast between the warm source and the environment. Rather, these data indicate that thermal IR sources in the range of human skin temperatures are most attractive to mosquitoes.

**Extended Data Fig. 4:**
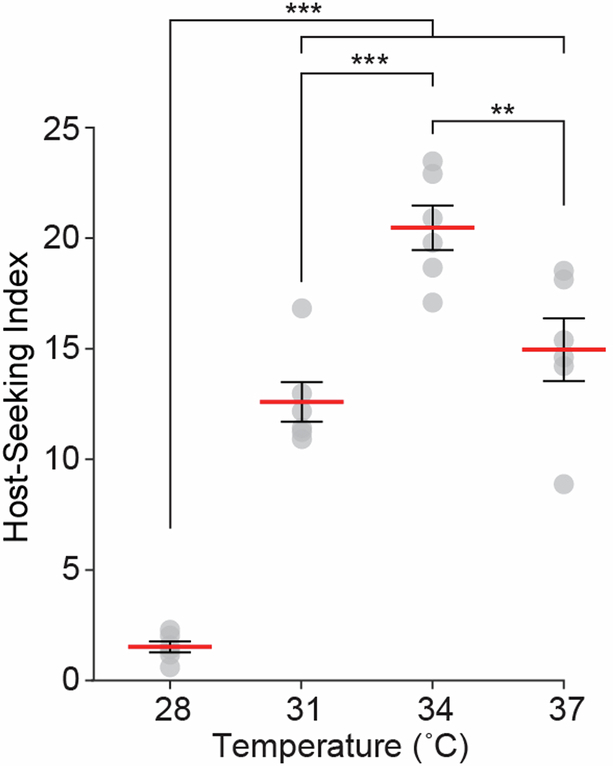
IR is an effective cue in single choice assays. Host-seeking response of the mosquitoes exposed to a single 34 °C IR source, 5% CO_2_ and human odour. Host-seeking index: average number of female mosquitoes host seeking throughout the five-minute experiment. Means ± SEMs (n = 6 biological replicates). One-way ANOVA with Tukey’s HSD test. **P < 0.01 and ***P < 0.001. Exact P-values provided in Source Data. Source Data

To test whether IR is an effective cue in a single-choice assay, we used a single Peltier device with a convective barrier similar to the two-way choice experiments and measured the HSIs when exposed to thermal IR across a range of temperatures (28 to 37 °C) in the presence of CO_2_ and human odour. We calculated the HSI in the Peltier zone and found that the HSI increased when the source of the thermal IR was between 31 °C and 37 °C, with a peak HSI at 34 °C (Extended Data Fig. 4).

## Flight behaviour drives shifts in PI

**Extended Data Fig. 5:**
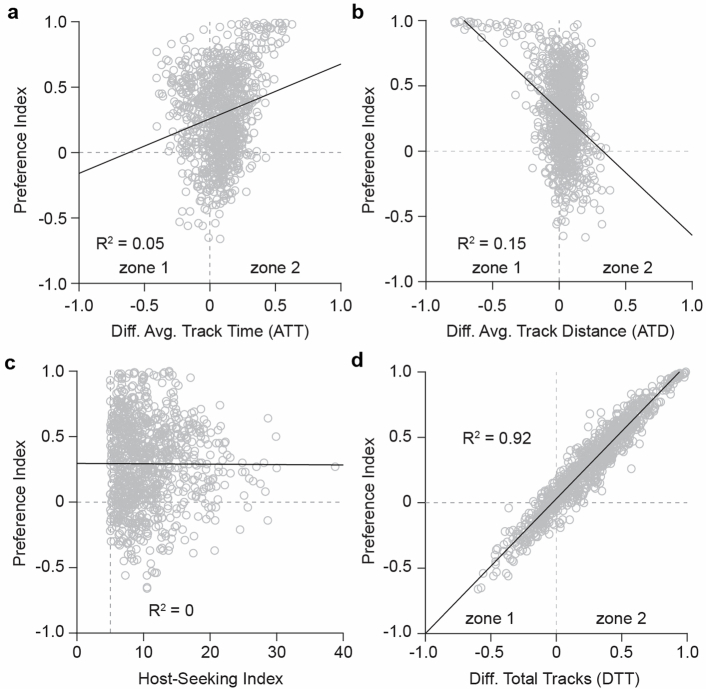
A correlation study of behaviours associated with shifting preference indexes. The data for these correlations were generated from 982 independent behavioural experiments (circles). Linear regression analyses were performed (black lines) and the fit of the models are indicated (R^2^). The average track time (ATT) for each zone is the cumulative host-seeking time spent in each zone divided by the overall number of tracks (bouts) observed in that zone. The average track distance (ATD) is the normalized average track distance difference between zones 1 and 2. The host-seeking index (HSI) is the average number of mosquitoes actively host seeking throughout the five-minute experimental window. The difference in total tracks (DTT) is the normalized difference in total number of tracks, ranging from −1 to 1. **a**, The correlation in ATT (where ATT = [ATT_zone2_ – ATT_zone1_]/[ATT_zone2_ + ATT_zone1_]) and recorded PI, R^2^ = 0.05 indicates no strong correlation between the preference index (PI) and the average time that the mosquitoes occupied that zone. The dashed lines cross the origin (0,0). **b**, The correlation in ATD (where ATD = [ATD_zone2_ – ATD_zone1_]/[ATD_zone2_ + ATD_zone1_]), and recorded PI, R^2^ = 0.15 indicates no strong correlation between the PI and the average distance that the mosquitoes occupied that zone. The dashed lines cross the origin (0,0). **c**, The correlation between the HSI, and recorded PI. R^2^ = 0; the vertical dashed lines indicate the HSI threshold value (HSI = 5); and the horizontal dashed lines indicate PI = 0. **d**, The correlation between the DTT (where DTT = [TT_zone2_ – TT_zone1_]/[TT_zone2_ + TT_zone1_] and recorded PI, R^2^ = 0.92 indicates a strong correlation between the preference index and the total number of tracks in each zone. This suggests that negative or positive PIs cannot be accounted for by behaviours exhibited after landing (ATT or ATD) or the overall host-seeking activity (HSI). Rather, the PI strongly correlated with the total tracks (DTT) recorded in each zone. Source Data

We illustrated above that the PI is a useful metric to summarize the overall preference of a group of mosquitoes throughout a given experiment. To determine whether pre-landing or post-landing behaviours were the primary drivers of the shifts in the PI, we analysed 982 individual behavioural experiments for correlations between the PIs and two post-landing behaviours. These include the average track time (ATT) and the average track distance (ATD; a detailed explanation of these metrics is provided in the Methods). We found that, after individual mosquitoes landed, there were no strong correlations between the PI and the average time that the mosquitoes occupied that zone (ATT; Extended Data Fig. 5a; *R*^2^ = 0.05) or the ATDs of the mosquitoes (Extended Data Fig. 5b; *R*^2^ = 0.15). We next investigated whether these same experiments had a correlation between the PI and the overall HSI (Extended Data Fig. 5c; *R*^2^ = 0) and found no correlation. These data indicate that strongly negative or positive PIs cannot be accounted for by behaviours exhibited after landing or the overall host-seeking activity. Rather, the PI strongly correlated with the difference in total tracks (DTT) recorded in each zone (Extended Data Fig. 5d; *R*^2^ = 0.92). Examination of these data suggests that the main factor that affects the PI metric is the overall number of mosquitoes that navigate to one zone versus the other. Our findings indicate that the preference for one zone over the other results from sensory integration and by decisions experienced before landing while the mosquitoes are in flight.

## IR is detected at mid-range

Convection heat can be detected only at close range (<10 cm)^16^. To determine the distance over which thermal IR can be detected by mosquitoes, we used a source of thermal IR that was 0.22 m^2^ (45.5 cm × 49 cm), which is a conservative size as it is less than the average surface area of an adult trunk facing a mosquito from the front or back (~0.28 m^2^; the estimate is shown in the Methods). We heated a 0.44 m^2^ plate (91 cm × 49 cm) to 34 °C and covered half of the plate with a 0.15-cm-thick clear acrylic panel to block IR transmission from that side (Fig. 2i). We applied human odour to the entire front surface of the cage mesh, and released 5% CO_2_ over the mesh surface from tubing mounted at the top of the cage. We also inserted a convective barrier, which consisted of high-IR-transmitting PE film 1 cm away from the cage to prevent convective cues from reaching the cage (Fig. 2i). We then measured the IR preference at set distances from the IR source. We found that *Ae. aegypti* displayed a strong preference for IR up to 70 cm (Fig. 2j; PI = 0.47 ± 0.04 at 30 cm; PI = 0.38 ± 0.04 at 70 cm). Beyond this distance, their preference for IR decreased substantially. At 80 cm, the PI was 0.15 and, at 90 cm, there was no preference (Fig. 2j; PI = 0.03 ± 0.06). Thus, we conclude that thermal IR is detected by *Ae. aegypti* at mid-range distances up to 0.7 m, which are much longer than the detection limit of convection heat from a 34 °C source (<10 cm), but not as long range as CO_2_, the most volatile human odours, and visual cues (up to around 5–15 m).

## IR sustains host seeking after CO_2_ pulse

**Fig. 3:**
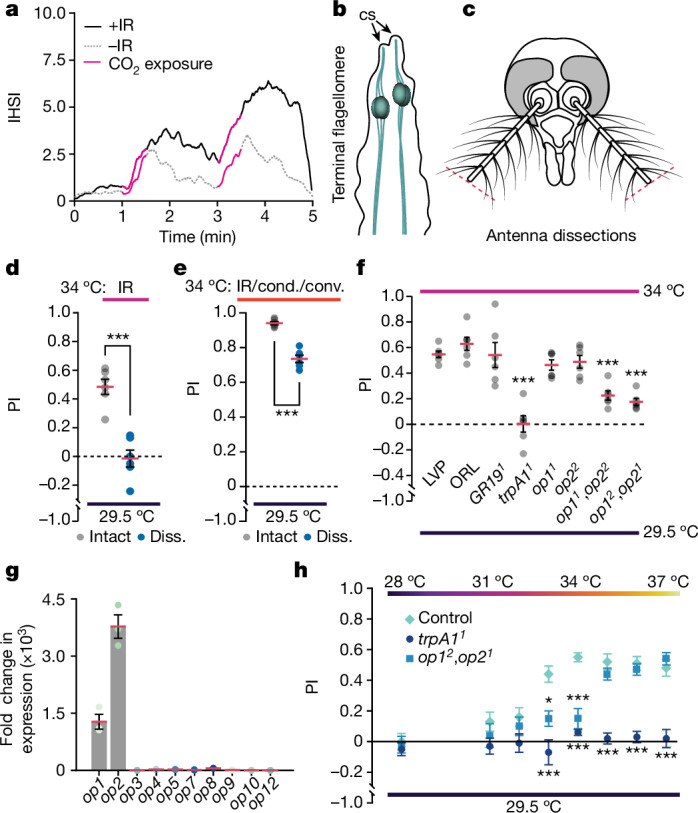
Molecular components of IR sensation*.* **a**,**d**–**f**,**h**, Females in both zones were exposed to 5% CO_2_ and human odour. Zone 1 was at ambient temperature (29.5 °C). Zone 2 was exposed to an IR source at 34 °C (**a**,**d**,**f**), all three forms of heat transfer at 34 °C (**e**) or an IR source only at the indicated temperature (**h**). **a**, The effect of CO_2_ on navigation towards IR on the basis of the IHSI. The average IHSI in the +IR and −IR zones represents the overall trend in host seeking at a specific time during CO_2_ exposure. **b**, Terminal flagellomere in an *Aedes* antenna. cs, coeloconic sensilla. Purported thermosensory neurons are shown in teal. **c**, The location of the dissection where the ends of the antennae were removed. **d**, IR-preference assays performed with dissected (diss.) and non-dissected (intact) wild-type (LVP) female mosquitos exposed to the presence or absence of IR. **e**, IR-preference assays performed with dissected and non-dissected LVP female mosquitos in the presence or absence of all three forms of heat transfer: IR, conductive (cond.) and convective (conv.) heat. **f**, Screen of mutant lines for impact on IR preference. LVP and Orlando (ORL) are the wild-type controls. **g**, qPCR analysis of all 10 *Aedes* opsin genes using RNA from female antennae. *n* = 3 biological replicates. Fold change in expression was calculated by normalizing to the lowest-abundance opsin, *op12*. **h**, The effects of the *trpA1*^*1*^ and *op1*^*2*^,*op2*^*1*^ mutations on IR preference across a range of Peltier temperatures. Data are mean ± s.e.m. Statistical analysis was performed using one-way ANOVA with Tukey’s multiple-comparison test (**f** and **h**) and parametric two-tailed Student’s *t*-tests (**d** and **e**); *P < 0.05. For **a**, **d**–**f** and **h**, *n* = 6 biological replicates for each group. Exact *P* values are provided as source data. Source Data

We showed that thermal IR promotes host-seeking activity, but only in the presence of other cues: human odour and CO_2_. Thus, mosquitoes must integrate IR sensation with these other stimuli. To examine the dynamics of this sensory integration, we modified the CO_2_ regime to score mosquito IR preference before and after two 30 s pulses of CO_2_. We measured each zone’s average instantaneous HSI (IHSI) across 18 trials (*n* = 6 biological replicates, 3 technical replicates each). The average IHSI indicates the overall trend in host seeking at a specific time during the CO_2_-exposure regime. Before the first CO_2_ exposure, the mosquitoes exhibited low overall host-seeking activity, and there was very little difference in the IHSIs in the presence and absence of IR (+IR and −IR, respectively; Fig. 3a). After exposure to CO_2_, there was a steep increase in the IHSI, which peaked after cessation of the CO_2_ stimulation, and then attenuated as the CO_2_ concentration in the local environment fell (Fig. 3a). Notably, in the presence of IR, mosquitoes demonstrated more sustained IHSIs after cessation of CO_2_ exposure compared with in the non-IR zone (Fig. 3a). In the absence of thermal IR, in response to the second CO_2_ pulse, there was only a slight increase in the HSI relative to the first CO_2_ pulse (Fig. 3a). However, in the presence of thermal IR, the second CO_2_ pulse caused the HSI to increase considerably over the first pulse (Fig. 3a). Together, our results indicate that attraction to IR is modulated by the presence of physiological levels of CO_2_.

## Thermal IR detection requires TRPA1

As the peak wavelength of the IR radiating from humans is far lower in energy than the longest wavelengths that mosquito rhodopsins can detect^36^, IR is unlikely to directly activate a receptor through stimulation by photons. An alternative explanation is that IR-sensitive structures in the mosquito undergo radiant heating, in turn activating a thermosensory receptor. According to this model, the IR energy radiating from the surface of the human body is absorbed by the mosquito, resulting in local heating.

The terminal segment of the mosquito antenna houses neurons associated with coeloconic sensilla that are warm-sensitive^12–15^ (Fig. 3b). Moreover, despite the reports concluding that *Aedes* do not respond behaviourally to IR^9,28,29^, there is some evidence that neurons in the terminal tip are stimulated by radiant heat^13^. The limited neuronal response to IR reported previously^13^ might be due to the use of a commercial IR emitter rather than a 34 °C source of radiant heat. Given how sensitive *Ae. aegypti* is to small differences in temperature (for example, 32 °C versus 33 °C; Fig. 2h), it is not surprising that a commercial IR emitter had only a small impact on the activation of these neurons.

To test whether mosquitoes require the distal part of the antennae for attraction to IR, we performed surgical dissections. We immobilized mosquitoes on ice and removed the distal half of each antenna using microscissors (Fig. 3c). Both the distal and proximal halves of the antenna are decorated with many olfactory sensilla, which house olfactory receptor neurons^37^. By removing only the distal portion of the antennae, we aimed to preserve some olfaction, while eliminating the region containing known thermosensory neurons^12–15^. We allowed the mosquitoes to recover for ≥2 days before performing the behavioural assays, and subjected control mosquitoes to the same anaesthetization and handling protocol, but left the antennae intact. We conducted all of the trials in the presence of elevated CO_2_ and human odour, and found that the dissections eliminated the preference for the zone exposed to IR from the 34 °C source (Fig. 3d; intact antenna, PI = 0.48 ± 0.05; dissected distal half, PI = −0.01 ± 0.06).

**Extended Data Fig. 6:**
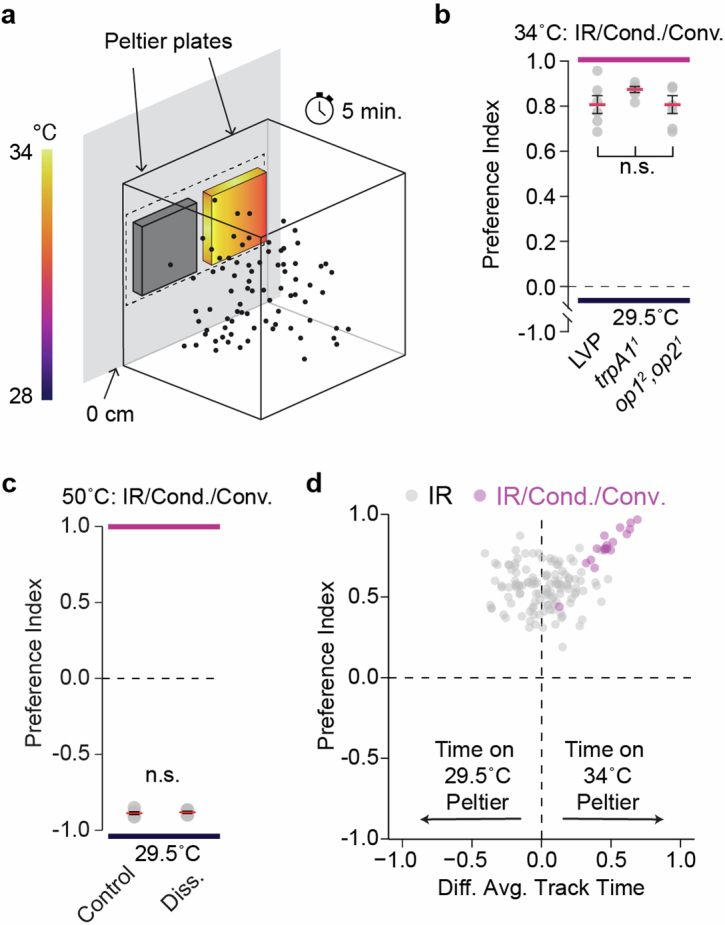
Effects of exposure of mosquitoes to all three modes of heat transfer. **a**, Setup to test the impact of all three modes of heat transfer on the selection of 34 °C versus ambient temperature (29.5 °C) in the presence of elevated CO_2_ and human odour. **b**, Testing effects of the indicated mutations on selecting the ambient temperature (29.5 °C), versus 34 °C under conditions in which the female mosquitoes are exposed to conductive, convective and radiant heat. Both zones were exposed to elevated CO_2_ and human odour. Means ± SEMs (n = 6 biological replicates). One-way ANOVA with Tukey’s multiple comparisons test. **c**, Preference assay of dissected (Diss.) versus non-dissected (Control) wild-type mosquitoes when exposed to all three forms of heat from a 50 °C source. Means ± SEMs (n = 6 biological replicates), two-tailed Student’s unpaired t-test. **d**, Dwell time of wild-type female mosquitoes exposed to IR versus IR with conductive and convective heat. Both zones were exposed to elevated CO_2_ and human odour. n.s., not significant. Exact P-values are provided in Source Data. Source Data

We then modified our set-up to allow the mosquitoes to be exposed to all three modes of heat transfer (conductive, convective and radiant) by placing the 34 °C and ambient temperature (29.5 °C) Peltier plates flush against the cage mesh (Extended Data Fig. 6a). As in our other experiments, both sides were also exposed to elevated CO_2_ and human odour. When we allowed mosquitoes to choose between 34 °C and 29.5 °C using this set-up, their preference for the 34 °C zone was very high (Fig. 3e). Moreover, after removing the distal half of the antenna, the preference for the 34 °C side was still robust, but reduced (Fig. 3e). We suggest that the moderate decrease in PI after the dissection (Fig. 3e) was due to the requirement of the distal end of the antenna for sensing IR. Thus, the distal half of the antenna is required for the detection of thermal IR (Fig. 3d), but is not essential for temperature discrimination at close range when all forms of heat transfer are available (Fig. 3e). This indicates that detection of conduction and/or convection heat is not solely dependent on the distal end of the antenna. These data also suggest that the avoidance of very high heat (≥50 °C) at close range, which involves all forms of heat transfer^17^, also does not require the end of the antenna.

The next question concerns a molecular explanation as to how *Ae. aegypti* females detect thermal IR. On the basis of our model, IR sensing requires a temperature sensor expressed in the distal region of the antenna. One candidate receptor is TRPA1, which is a known warm sensor in fruit flies and mosquitoes^15,17,38–41^. Another potential receptor is GR19—the *Aedes* homologue of *Drosophila* GR28b^17^, which in the fruit fly also functions as a thermosensor^42^. We therefore compared the PI values of the *Gr19*^*1*^ and the *trpA1*^*1*^ mutants with the Liverpool (LVP) wild-type strain that we characterized in the preceding analyses, as well as the Orlando (ORL) wild-type strain. We found no significant difference between the PI values exhibited by the two wild-type strains (Fig. 3f; LVP, PI = 0.55 ± 0.03; ORL, PI = 0.63 ± 0.05). Similarly, the *Gr19*^*1*^ mutant line displayed a robust preference for the IR zone, similar to the wild type (Fig. 3f; 0.54 ± 0.10). By contrast, IR preference was eliminated in *trpA1*-mutant females (*trpA1*^*1*^; Fig. 3f; PI = 0.00 ± 0.06).

When we used the modified set-up that allows the mosquitoes to be exposed to all three forms of heat transfer (conductive, convective and radiant; Extended Data Fig. 6a), both the wild-type and the *trpA1*^*1*^ mutant showed the same preference for the 34 °C zone (Extended Data Fig. 6b). These findings are consistent with a previous study showing that, if the mosquitoes are exposed to all modes of heat transfer at close range, then *trpA1* is required only for sensing very high temperatures (>50 °C)^17^. This *trpA1*-dependent sensation of >50 °C is not mediated through neurons at the end of the antenna as, when we removed the distal part of the antenna and exposed the mosquitoes to a 50 °C Peltier device flush up against the cage mesh, both the dissected and intact control mosquitoes avoided landing on the 50 °C Peltier plate surface to the same extent (Extended Data Fig. 6c; PI = −0.88 ± 0.01 (dissected); PI = −0.89 ± 0.01 (intact control)). We then compared the dwell times of wild-type females on the 34 °C and 29.5 °C zones to determine whether there is any difference depending on whether the mosquitoes were exposed to all three modes of heat transfer or just IR. We found that the dwell time increased significantly when the females were exposed to conduction, convection and IR, relative to IR only (Extended Data Fig. 6d).

**Fig. 4:**
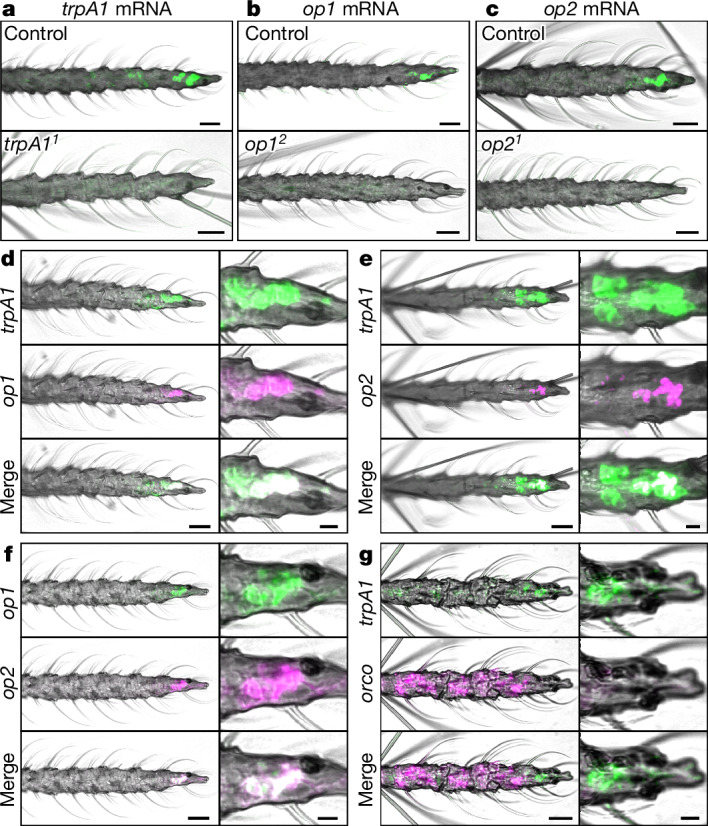
Expression of genes involved in IR sensation. In situ hybridization analysis of *trpA1*, *op1*, *op2* and *orco* mRNAs at the female antennal tip (13th flagellomere). **a**, Probing for *trpA1* mRNA in the wild-type control and the *trpA1*^*1*^ mutant. **b**, Probing for *op1* mRNA in the wild-type control and the *op1*^*2*^ mutant. **c**, Probing for *op2* mRNA in the wild-type control and the *op2*^*1*^ mutant. For **a**–**c**, *n* ≥ 5. **d**–**g**, Double in situ hybridizations in wild-type antennae. Co-localizations of the signals are shown in white in the merged subpanels. **d**, In situ hybridization analysis of *trpA1* (green) and *op1* (magenta). **e**, In situ hybridization analysis of *trpA1* (green) and *op2* (magenta). **f**, In situ hybridization analysis of *op1* (green) and *op2* (magenta). **g**, In situ hybridization analysis of *trpA1* (green) and *orco* (magenta). For **d**–**g**, *n* = 3. Scale bars, 20 μm (full views) and 5 μm (insets).

**Extended Data Fig. 7:**
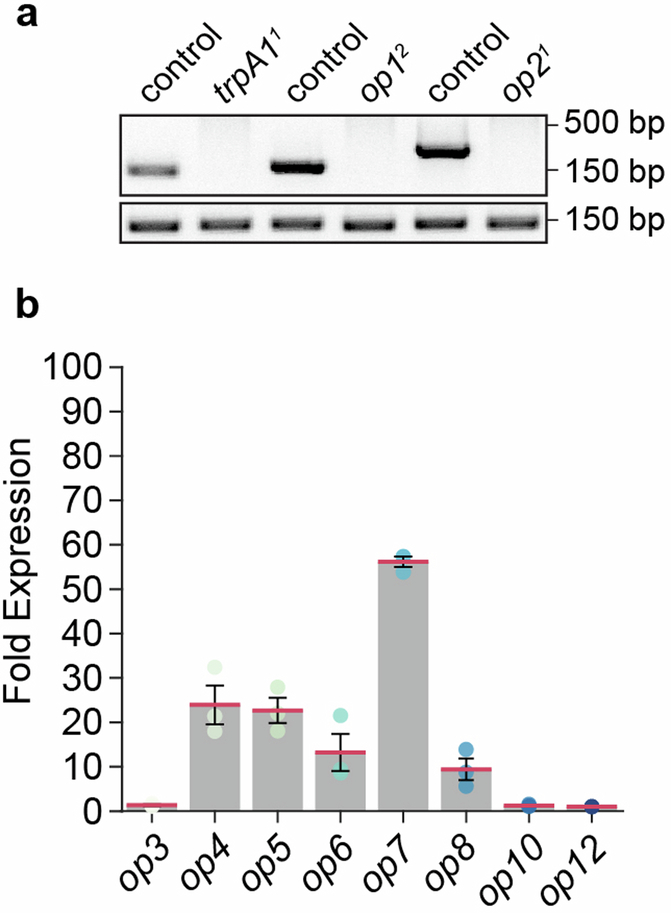
Expression of *opsin* and *trpA1* RNAs in antennae assayed by RT-PCR. **a**, RT-PCR with primers specific to *trpA1*, *op1*, and *op2* mRNAs using cDNA derived from female antennae. Primers specific to *RpL17* mRNA were used as a positive control (bottom row). RNAs from the control and mutant mosquitoes were used to generate the PCR products for the indicated genes (*trpA1*, *op1* and *op2*) and for *RpL17*. The PCR products for *trpA1*, *op1* and *op2* and for *RpL17* were loaded in adjacent sets of wells on the same 1% agarose gel. The original scan of the experiments from two biological replicates are shown in Supplementary Fig. 3. **b**, Expanded qPCR data (from Fig. 3g) for the low-abundance opsin genes. n = 3 biological replicates, Means ± SEMs. Source Data

To determine whether *Aedes* TRPA1 is expressed in the distal region of the antenna, which houses thermosensory neurons, we analysed expression of *trpA1* mRNA. First, we performed PCR with reverse transcription (RT–PCR) using cDNA derived from female antennal tissue and detected *trpA1* transcripts from the wild-type control but not the *trpA1*^*1*^ mutant (Extended Data Fig. 7a). Second, to determine whether *trpA1* is expressed near the tip of the antenna, we spatially localized the transcripts using in situ hybridization, and detected *trpA1* mRNA at the distal end of wild-type antennae (Fig. 4a).

## Two opsins function in IR detection

In *Drosophila*, TRPA1 is also a sensor of warm temperatures and functions in avoiding noxious temperatures^38,39,43^. Moreover, *Drosophila* TRPA1 is required for discriminating between different comfortable temperatures that are below the threshold for direct activation of the channel^44^. Thus, in the comfortable range, TRPA1 is not the direct temperature sensor. Rather, to sense comfortable temperatures, several opsins are also required for thermosensation, and they initiate a signalling cascade that culminates in the activation of TRPA1^45,46^.

We wondered whether one or more *Aedes* opsins might be required for sensing thermal IR, but only at lower levels of radiant heat intensity due to lower temperatures. A prime candidate opsin would be one that is expressed in the antenna. We therefore isolated total RNA from female antennae, prepared cDNA and performed quantitative PCR (qPCR) analysis using primers specific to each of the ten *Aedes* opsin genes. We found that two opsin genes, *GPROP1* (*op1*) and *GPROP2* (*op2*), were expressed in the antenna (Fig. 3g), while the other eight were either undetectable, or expressed at far lower levels (Fig. 3g and Extended Data Fig. 7b). Moreover, we confirmed the absence of *op1* and *op2* transcripts in the *op1*^*2*^ and *op2*^*1*^ mutants, respectively, using RT–PCR (Extended Data Fig. 7a).

To determine whether *op1* and *op2* mRNAs are present in the terminal segment of the antenna, we performed in situ hybridization analysis. We found signals for both *op1* and *op2* in neurons in the most distal region of the antenna (Fig. 4b,c). To examine whether *op1*, *op2* and *trpA1* are expressed in the same cells in the antennae, we carried out in situ hybridizations using each permutation of probes against two out of the three genes. We found that *op1* and *op2* co-localized with *trpA1* (Fig. 4d,e) and with each other (Fig. 4f) near the distal tip of the antenna. Moreover, some cells proximal to these neurons also expressed *trpA1* but not *op1* or *op2* (Fig. 4a–e). The vast majority of sensory hairs in the antennae house olfactory receptor neurons, most of which are labelled by the odorant co-receptor (ORCO)^47^. However, we did not detect co-labelling of *trpA1* and *orco* in the distal region of the antenna (Fig. 4g), suggesting that these *trpA1*-positive neurons do not function in olfaction.

**Extended Data Fig. 8:**
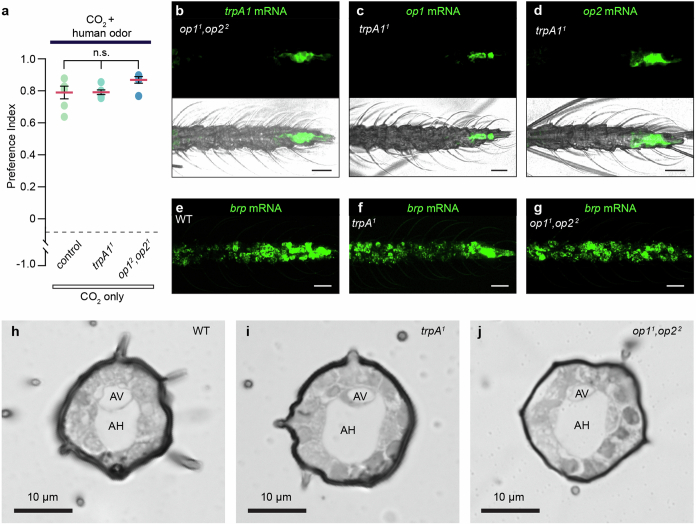
Checking whether the *trpA1* and *opsin* double mutants display normal taxis, and presence of *trpA1*- and *opsin*-expressing neurons in sensilla at the distal end of the mutant antennae. **a**, Odour preference assays performed on control, *trpA1*^*1*^, and *op1*^*2*^,*op2*^*1*^ in response to CO_2_ alone versus CO_2_ with human odour. Each group is n = 6 (biological replicates). Means ± SEMs. One-way ANOVA with Tukey’s test for multiple comparisons. n.s., not significant. **b**-**g**, In situ hybridizations at the distal end (13^th^ flagellomere) of female antennae. Confocal images below in **b**-**d**. **b**, *trpA1* mRNA in the *op1*^*2*^,*op2*^*1*^ mutant. **c**, *op1* mRNA in the *trpA1*^*1*^ mutant. **d**, *op2* mRNA in the *trpA1*^*1*^ mutant. n ≥ 5 for b-d. **e**, *brp* mRNA in WT. **f**, *brp* mRNA in the *trpA1*^*1*^ mutant. **g**, *brp* mRNA in the *op1*^*2*^,*op2*^*1*^ mutant. n = 3 for e-g. **a**-**g**, Scale bar is 20 μm. **h**-**j**, Cross-sectional bright-field images of the distal end (13^th^ flagellomere) of female antennae. Antennal vessel (AV), antennal haemocoel (AH), Scale bars are 10 μm. **h**, WT. **i**, *trpA1*^*1*^ **j**, *op1*^*2*^,*op2*^*1*^. n = 3 for h-j. Exact P-values provided in Source Data. Source Data

The co-expression of *op1* and *op2* with *trpA1* raises the possibility that one or the other opsin functions in sensing thermal IR. However, the individual *op1* and *op2* mutants exhibited only slight reductions in preferences for IR that were not statistically significant (Fig. 3f; PI = 0.46 ± 0.04 and PI = 0.44 ± 0.05, respectively). We therefore tested a double mutant (*op1*^*1*^,*op2*^*2*^) and found that it had a significant defect in IR preference, although the preference for IR was not eliminated (Fig. 3f; PI = 0.23 ± 0.04). The phenotype of a second double mutant (*op1*^*2*^,*op2*^*1*^) was the same (Fig. 3f; PI = 0.18 ± 0.01). The deficits in IR sensation in the *trpA1* and *opsin* double mutants were not due to an inability to direct their movements or due to a deficit in visually detecting the zones, as these mutant mosquitoes were as effective as the wild type at navigating to a zone with human odour and CO_2_ over a zone exposed to CO_2_ only (Extended Data Fig. 8a). To conduct these experiments, no thermal IR source was used (both Peltier devices were at ambient temperature) and human odour was applied to only one half of the front cage mesh. Moreover, neither the *trpA1* nor the *op1*,*op2* mutants eliminated the neurons at the distal end of the antenna as *trpA1* RNA was expressed in the *op1*,*op2* mutant, and the *op1* and *op2* RNAs were expressed in the *trpA1* mutant antenna (Extended Data Fig. 8b–d). We counted the number of neurons in the terminal (13th) flagellomere of the antenna by staining for *bruchpilot* (*brp*) RNA, which is expressed pan-neuronally^48^, and analysed the *z* stacks using confocal microscopy. There was no apparent loss of total neurons in the 13th flagellomere of the antenna of the *trpA1* (90 ± 15) or *op1*,*op2* (86 ± 15) mutants relative to the wild type (85 ± 13; Extended Data Fig. 8e–g). We also examined sections from the tip of the 13th flagellomere of wild-type and *trpA1*- and *op1*,*op2*-mutant females and found that the gross morphologies were similar, including the antennal vessel and antennal haemocoel running through the antennal cavity^49^ (Extended Data Fig. 8h–j). These data indicate that the behavioural phenotypes exhibited by the *trpA1* and *op1*,*op2* mutants were not due to loss of neurons or to an obvious morphological deficit.

Opsins initiate signalling cascades that are very effective in signal amplification^50^. We therefore set out to test the model that Op1 and Op2 would be required to sense thermal IR only at the lower intensities, while TRPA1 would be required for sensing thermal IR at both lower and higher intensities. According to this model, the more-intense levels of IR would generate sufficient heating to directly activate TRPA1. However, at lower levels of radiant heat, the opsins would be required to initiate an amplification cascade that culminates in the activation of TRPA1. Consistent with this proposal, *trpA1* was required at all intensities of radiant heat, while *op1* and *op2* were required only for sensing the lower levels (Fig. 3h). The normal IR responses of the *op1*,*op2* mutants in response to thermal sources of ≥35 °C also support the conclusion that the deficits in sensing thermal IR from a 33 °C or a 34 °C source is not due to an impairment in vision.

## TRPA1/opsins sense heat at antennal end

**Fig. 5:**
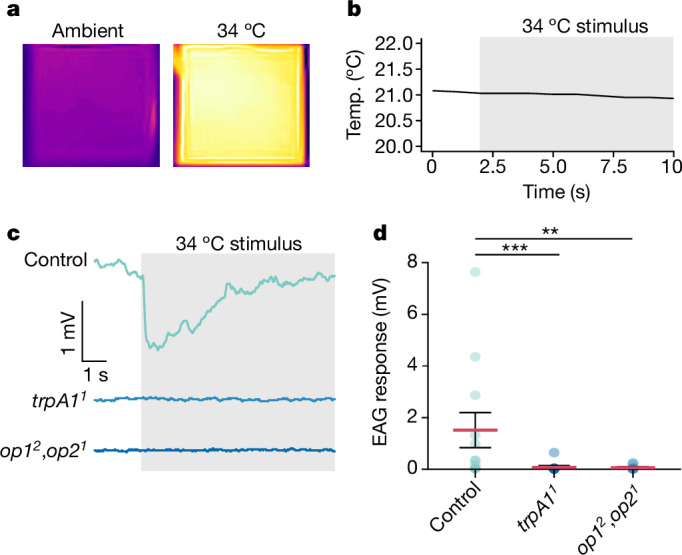
EAG analysis to address whether heat activation is impaired in the *trpA1* and *opsin* mutants. **a**, IR thermograph of the thermal output of the 4 cm × 4 cm × 1 cm IR source used in the experiments. Right, the 4 cm × 4 cm × 1 cm block was held at around 34 °C. Left, the same block at ambient temperature (around 21 °C). The images were acquired using the FLIR One IR smartphone camera. **b**, The temperature at the site of the antennal preparation before exposure to the 34 °C block and during exposure to the 34 °C IR stimulus (grey shading). The temperature was measured by placing the tip of the probe (TSP01-USB Temperature and Humidity Data Logger; Thor Labs) at the same site as the antennal preparation used for the EAG experiments. **c**, Representative EAG traces from wild-type (control), *trpA1*^*1*^ and *op1*^*2*^,*op2*^*1*^ female mosquitos that were presented with the IR source (grey shading). **d**, EAG amplitudes exhibited by wild-type (control), *trpA1*^*1*^ and *op1*^*2*^,*op2*^*1*^ mosquitos in response to a 34 °C IR source. *n* = 12 (wild-type control and *trpA1*^*1*^) and *n* = 11 (*op1*^*2*^,*op2*^*1*^). Each datapoint represents EAG responses from individual mosquitoes. Data are mean ± s.e.m. Statistical analysis was performed using one-way aligned-rank ANOVA with pairwise contrasts (**d**); **P < 0.01. Exact *P* values are provided as source data. Source Data

Our data indicate that the opsins and TRPA1 function as heat sensors at the distal end of the antennae. To test this model, we performed electroantennograms (EAGs) using antennae exposed to IR from a 34 °C source. To deliver the IR stimuli, we circulated hot water from a 37 °C bath through a 4 cm × 4 cm × 1 cm aluminium plate covered with white ConTact paper (emissivity, 0.92) so that the final surface temperature was 34 °C (Fig. 5a). The stimulus was applied by moving the heated plate to 3 cm from the mosquito antennae. From this distance, the air temperature at the position of the mosquito antenna did not increase above the ambient temperature (around 21 °C) when it was exposed to the 34 °C source of IR (Fig. 5b). Thus, the antenna did not receive convection heat from the IR source. To ascertain that the mosquitoes were responsive, we recorded EAG responses elicited by a positive control (mouth puff; Methods). Any mosquito that did not show an EAG response to the positive control before the heat stimulus was excluded. If there was no response to the heat stimulus, we performed a second positive control afterwards to confirm that the antenna was responsive. We found that the 34 °C stimulus elicited an EAG response in most (8 out of 12) wild-type control females (Fig. 5c,d). By contrast, nearly all of the *trpA1*^*1*^ and *op1*^*2*^,*op2*^*1*^ mutant mosquitoes did not display responses (Fig. 5c,d). The one exception for *trpA1*^*1*^ (1 out of 12) showed only a moderate response (Fig. 5d; 0.64 mV), and the two exceptions for *op1*^*2*^,*op2*^*1*^ (2 out of 11) elicited very small responses (Fig. 5d; 0.20 and 0.25 mV). The response elicited by the control was due to thermal IR, as there was no conductive or convective heat transferred in these experiments.

## Discussion

The finding that thermal IR is an important cue that *Ae. aegypti* females use to find their targets counters reports that *Ae. aegypti* do not respond behaviourally to thermal IR^9,28,29^. However, previous research examined IR in isolation. *Ae. aegypti* require multisensory integration to home in on people, and individual human-associated cues, such as IR, CO_2_ plumes and organic odours, have little efficacy in stimulating host-seeking behaviour on their own^3–6^. Our finding that IR is used in combination with other cues adds critical breadth to the *Aedes* toolkit—allowing them to home in on humans from mid-range distance in varied and dynamic environments.

The thermal IR that emanates from surface body temperature is far lower in energy than the longest wavelengths that activate visual pigments^20^. Rather than detecting photons directly, a more plausible mechanism for thermal IR detection is that the radiant energy warms dendrites in coeloconic sensilla near to the tip of the antenna, which in turn activates thermosensitive receptors. In support of this model, removal of the distal portion of the antenna, which contains heat-sensitive neurons^12,13,15^, eliminates IR attraction. While past research reports that the coeloconic sensilla at the distal end of the antenna sense convective heat^13^, the design of the thermosensitive peg-in-pit coeloconic sensilla at the antennal tip is more consistent with a sensor of radiant than of convective heat. Located in a pit, the neurons would be largely protected from convective currents, and would receive radiant heat preferentially from the direction of the pit aperture. Directionality is important for a radiant heat sensor, but would reduce the sensitivity of a convective heat sensor. Nevertheless, at very close range, the neurons in peg-in-pit sensilla would also be activated by convection heat.

We propose that IR impinging on the peg-in-pit sensilla is partially absorbed by the cuticle, and then energy is transferred to the endolymph through conduction. Some IR might also penetrate the cuticle and directly warm the endolymph. We found that the heat-activated TRPA1 channel is expressed in neurons at the antennal tip and is required for responding to IR. By contrast, *trpA1* mutants display normal attraction to conductive/convective heat in the temperature range of human skin^17^. In the presence of conductive/convective heat, the role for TRPA1 is to help to avoid ≥50 °C conditions (ref. ^17^), even though TRPA1 is activated with a threshold of only around 32 °C (ref. ^41^). The anatomical location for this close-range TRPA1 function appears to be distinct from IR sensation. Our data support the model that TRPA1 senses IR at mid-range distances (~0.7 m) through the ‘warming neurons’ in the peg-in-pit sensilla.

Two opsins (*op1*, *op2*) and *trpA1* are co-expressed at the end of the antenna, and mutations eliminating these opsins reduce IR sensation, but only at lower intensities of radiant heat. We propose that contributions of both opsins and TRPA1 to detecting radiant heat endows mosquitoes with a greater dynamic range for sensing radiant heating. We suggest that, at higher IR intensities, the radiant heat is sufficient to directly activate TRPA1, while, at lower levels of thermal IR, activation of the opsins initiates a cascade that amplifies the signal and indirectly activates TRPA1.

In conclusion, thermal IR represents an important mid-range cue that is used by *Ae. aegypti* to couple longer- and shorter-range cues. As *An. stephensi* are also attracted to thermal IR, we speculate that detection of the IR may be widely used among blood-feeding mosquitoes to home in on warm-blooded hosts. Finally, the finding that thermal IR is an effective host-seeking cue raises the possibility of developing strategies to interfere with this attraction, and the opportunity to devise more effective mosquito baits (Supplementary Discussion).

## Methods

### Mosquito stocks

The *Ae. aegypti* Liverpool strain (LVP) was provided by O. Akbari (UCSD) and was used as the wild-type strain in this study. The *trpA1*^*1*^ (referred to as *trpA1*^*−/−*^ and *trpA1*^*ECFP*^ in ref. ^17^), *Gr19*^*1*^ (referred to as *Gr19*^*−/−*^ and *Gr19*^*DsRed*^ in ref. ^17^) and Orlando (ORL) *Ae. aegypti* strains were provided by L. Vosshall (Rockefeller). The *trpA1*^*1*^ line was outcrossed to the LVP line for five generations and a homozygous line was regenerated. We previously described the *op1*^*1*^, *op1*^*2*^, *op2*^*1*^and *op2*^*2*^ strains, their double-mutant versions (*op1*^*1*^,*op2*^*2*^ and *op1*^*2*^,*op2*^*1*^) and outcrossing of these lines^51^. Since this publication, we have adopted a new nomenclature for naming mutant and transgenic lines. The updated and previous allele names, respectively, are: *op1*^*1*^ = *op1*^*R*^, *op1*^*2*^ = *op1*^*G*^, *op2*^*1*^ = *op2*^*R*^, *op2*^*2*^ = *op2*^*G*^. The *An. stephensi* strain was obtained from O. Akbari (UCSD). All mosquitoes for each line were randomly selected for analysis at 1–3 weeks of age. We used females exclusively, because only females display host-seeking behaviour.

### Mosquito rearing and maintenance

Mosquitoes were raised in 28 °C chambers with 80% relative humidity under 14 h–10 h light–dark cycles. Mosquito larvae were hatched in reverse osmosis water and reared using fish food (TetraMin Tropical Granules, 16122, Tetra). Adult mosquitoes were maintained on a 10% sucrose (w/v) solution. For propagating mosquitoes, females were blood fed on a membrane feeding system (SP6W1-3, HemoTek) containing defibrinated sheep blood (DSB250, HemoStat Laboratories). The rearing and maintenance procedure was approved and monitored by the Institutional Animal Care and Use Committee at UCSB.

### Set-up to conduct IR-preference assays

The arena used for behavioural assays was custom fabricated by the UCSB Physics Machine Shop. Five panels of 0.5 inch acrylic (8560K268, McMaster-Carr) were machined as described in Extended Data Fig. 1a. Two 10 cm × 10 cm cutouts were made in one panel with enough tolerance to hold the two Peltier plates (10 cm × 10 cm) securely (TEC plate, model TCP-50, Advanced Thermoelectric). The panels were assembled using stainless steel socket head screws (McMaster-Carr). An LED light bar was mounted to illuminate the wall that contained the Peltier devices (B07CVCF8JF, YEEZEN).

All but one of the interior faces of the arena panels were covered in white PVC adhesive (ConTact, Kittrich) paper to limit unwanted visual stimuli in the mosquito visual field and produce high contrast images (dark mosquito bodies versus light background) for subsequent object tracking. The Peltier plates were also covered with white ConTact paper (emissivity, 0.92). Moreover, the Peltier devices housed in the arena wall were recessed from the interior face of the arena by 0.5 cm to provide an air gap when it covered (Extended Data Fig. 1b). Behind the panel that was left clear, we mounted a webcam (Logitech c920, Logitech) trained on the arena wall that housed the Peltier plates (Extended Data Fig. 1b). Experiments were video recorded using the Logitech Webcam Software (v.2.51).

The arena was designed to accommodate 30 cm × 30 cm × 30 cm mosquito cages (BugDorm-1, DP1000, MegaView Science) placed inside the arena from the top. To improve the imaging quality, we replaced one mesh panel of the cage with a 1/16 inch thick, clear acrylic panel (8560K171, McMaster-Carr) (Extended Data Fig. 1c), and these are referred to as assay cages throughout this study. This modification enabled us to achieve a sharper, higher-contrast image, which improved our tracking ability. The clear acrylic panel was held in place with small machine nuts and bolts (92000A155, 91828A220, McMaster-Carr). The side of the cage facing the Peltier thermal targets was covered with fine polypropylene netting, which is highly transparent to IR.

An automated CO_2_-release system was constructed using a 12 V Electric Solenoid Air Valve (KL04010, BEDUAN) that was controlled by an Uno R3 Controller Board (EL-CB-001, ELEGOO) and a 12 V Relay Module (HiLetgo). The controller board was programmed with a custom Arduino script that opened and closed the solenoid valve at predetermined intervals. The CO_2_ was released in the arena through perforated tubing surrounding the Peltier plates (Extended Data Fig. 1d). To measure the CO_2_ dynamics during a typical experimental regime, we placed a CO_2_ sensor (CO_2_ Meter Gas Measurement Specialists, 030-8-0010, K30 FR fast response 10,000 ppm CO_2_ sensor) inside the assay cage on either the left or right-hand side directly centred in each zone. We measured the increase in local CO_2_ concentration for 5 min using GasLab software. We found that the absolute CO_2_ concentrations ranged from 500 to 800 ppm when recorded from a CO_2_ sensor placed inside an assay cage and exposed to the 5 min CO_2_ paradigm used in the study. We found no difference in left/right CO_2_ concentration (Extended Data Fig. 1e). This system allowed for the reproducible release of CO_2_ at set intervals across experimental replicates. Wiring schematics and code are available on request.

Human odour was applied by wiping a worn glove across the entire front surface of the cage mesh 5–10 times. As the temperature of the surface of both sides of the cage was the same (Extended Data Fig. 2a), increased odour volatility due to a higher temperature on one side is not an issue. In Extended Data Fig. 8a, human odour was applied as described above to only one half of the front cage mesh. To conduct these experiments, no thermal IR source was used (both Peltiers were at the ambient temperature of 29.5 °C).

### Behavioural assays for measuring responses to IR

Unless indicated otherwise, 80 female mosquitoes were manually aspirated from the grouped rearing cage and placed in modified experimental cages the day before experimentation, where they were allowed to feed on 10% sucrose ad libitum. The age of the mosquitoes used for the behavioural experiments was 1–3 weeks. Most of the experiments were conducted in the subjective morning (zeitgeber time 1–5), during which the endogenous host-seeking activity is high (Supplementary Fig. 1a). We used larger numbers of mosquitoes for the mock-antenna-dissected and distal-antenna-dissected groups (175 and 300, respectively) to achieve HSIs in the 5–20 range, as these mosquitoes exhibited an overall reduction in host-seeking activity.

For a single given condition, each cage (biological replicate, *n*) was tested a minimum of three times (technical replicates). If replicates met inclusion criteria, they were averaged and each average was used to calculate the mean response of that cage (*n*). If a cage did not achieve two suitable replicates, it was not included in this study. For the experiments in Extended Data Fig. 8a, we did not use an HSI criterion of 5 because CO_2_ + odour elicits a weaker HSI than CO_2_, odour and IR together.

### Testing behaviour using the set-up with a second PE film 2 cm from cage mesh

To provide an additional test to verify that convective warming from the 34 °C Peltier is not reaching the surface of the mosquito cage, we modified the set-up described above (see the ‘Set-up to conduct IR-preference assays’ section) and added a second PE film 2 cm from the first PE layer and 2 cm away from the cage mesh where mosquitoes land. The temperature was recorded at the surface of the cage mesh and at the surface of the second PE film over a 5 min time span using a temperature probe (TSP01-USB Temperature and Humidity Data Logger; Thor Labs). Preference assays were performed with wild-type (LVP) females using this modified set-up in which we exposed one side to IR from a 34 °C source, and both sides were exposed to human odour and 5% CO_2_.

### Testing response to IR using a one-way choice assay

To test whether IR is an effective cue in a one-way choice assay we modified our set-up (see the ‘Set-up to conduct IR-preference assays’ section) and used a single Peltier device with a convective barrier similar to the two-way choice experiments. The behavioural assays were performed with wild-type (LVP) female mosquitoes across a range of Peltier temperatures (28−37 °C) in the presence of CO_2_ and human odour. The HSI was calculated as the average number of female mosquitoes host seeking at the cage’s mesh directly opposite to the single Peltier device throughout the 5 min experiment.

### Set-up for measuring effective distance for detecting IR

To assay the distance over which female mosquitoes are able to detect thermal IR, we first took into consideration that the average surface area of the front of an adult human trunk is ~0.28 m^2^. This is based on an estimate that the total surface area of an average human is 1.7 m^2^ (https://www.calculator.net/body-surface-area-calculator.html), that the trunk consists of around 36% of the total surface area^52^ and that approximately 45% of the trunk faces the mosquito from the front or back. To conduct our experiments, we used an IR source that was 0.22 m^2^ (45.5 cm × 49 cm), which is a conservative estimate of the area of an IR source available to a mosquito. We covered a 0.44 m^2^ (91 cm × 49 cm) electric plate made out of stainless steel (Hatco, GRS-36-I) with white ConTact paper (emissivity, 0.92), and then heated the plate to 34 °C. To block IR from half of the plate, we placed a clear acrylic panel 10 cm in front of one half of the plate (Fig. 2i). To prevent convection heat from the 34 °C IR surface reaching the mosquito cage that we used to perform the behavioural assays, we placed a high-IR-transmitting PE film in front of the cage. An acrylic panel was placed as a barrier between the IR and non-IR sides to prevent any bleed-through of IR radiation to the non-IR side. The CO_2_ was released at the IR-facing surface of the cage through perforated tubing attached to the top of the front panel of the cage (Fig. 2i). Human odour was applied by wiping a worn glove across the entire front surface of the cage mesh 5–10 times.

To conduct the behavioural assays, we transferred 80 female mosquitoes into the assay cages and allowed them to acclimatize for a minimum of 24 h, while they were allowed to feed on 10% sucrose ad libitum. We then placed the cage at different distances from the IR surface and monitored host-seeking behaviour for 5 min by recording movement after landing of mosquitoes on the mesh facing the IR and non-IR sides in the presence of human odour and 5% (v/v) CO_2_.

### Set-up to assay responses to all three modes of heat transfer from a 34 °C source

To modify our set-up to allow the mosquitoes to be exposed to all three forms of heat transfer (conductive, convective and radiant) from a 34 °C source, we removed the convective barrier (PE) and situated two Peltiers (one at 34 °C, and one at 29.5 °C) flush against the cage mesh, therefore allowing all forms of heat transfer to be present in the assay. To conduct the behavioural assays, we transferred 80 female mosquitoes into the assay cages and allowed them to acclimatize for ≥24 h, during which they were allowed to feed on 10% sucrose ad libitum. We then placed the cage inside the arena and monitored their host-seeking behaviour for 5 min by recording movement after landing on the cage’s mesh flushed against the Peltier devices in the presence of human odour and 5% (v/v) CO_2._

### Set-up to assay responses to all three modes of heat transfer from a 50 °C source

To expose the mosquitoes to all three forms of heat from a 50 °C Peltier source, we modified the set-up (see the ‘Set-up to conduct IR-preference assays’ section) by removing the convective barrier (PE) and placing the single Peltier device set at 50 °C flush against the cage mesh. This allowed the mosquitoes to be exposed to all three forms of heat transfer: convection, conduction and radiant. The behavioural assays were performed in the presence of CO_2_ and human odour. Preference assays were performed in the modified set-up described directly above with either intact wild-type (LVP) females, or with female mosquitoes in which the distal ends of the antennae were removed. We tested the aversion of the mosquitoes to 50 °C in a conduction/convection/IR setting (landing on plate) where the plate was 50 °C and the cage surface was exposed to human odour and 5% CO_2_. The PI of the mosquitoes landing on the 10 × 10 cm 50 °C Peltier plate (zone 1) versus landing on the surrounding area of the same size (100 cm^2^; zone 2) was calculated (PI = HSI of zone 1 − HSI of zone 2/HSI of zone 1 + zone 2).

### Air temperature recordings

A temperature probe (TSP01-USB Temperature and Humidity Data Logger, Thor Labs) was positioned inside the behaviour cage 4 cm away from the wall of the behaviour arena, directly opposing the Peltier plates. With the convective barrier either in place or removed, we recorded air temperatures for 5 min under various conditions. We measured the air temperature in front of the Peltier set to a range of temperatures (28−37 °C) as well as the control Peltier (turned off, equilibrated to ambient conditions). Moreover, we shielded the temperature probe with aluminium foil to prevent incident IR from heating while still allowing ample air space around the probe to allow for measurements. We therefore calculated the mean temperature value as well as the minimum and maximum recorded values (Extended Data Fig. 2a,b).

### Quantifying IR using a pyroelectric detector

To measure IR levels across different Peltier temperatures, and different distances from the Peltier, we used a DLaTGS (deuterated l-alanine doped triglycine sulphate) type pyroelectric IR detector (Bruker, D301) featuring a potassium bromide window with a spectral range spanning 1–25 μm and a sensor surface area of 1 mm^2^. The signal from the IR source was modulated through an optical chopper at 20 Hz (Scitec Instruments, 340CD), and the signal was measured with a lock-in amplifier (Stanford Research Systems, SR530).

To measure the IR signal from the Peltier device at different temperatures (28 °C, 31 °C, 34 °C and 37 °C), we initially constructed a standard plot of black body radiation emitted from black insulation foam at varying temperatures. The total energy emitted by the black body surface was determined using the Stefan–Boltzman law. Subsequently, we computed the energy at the sensor by considering the inverse square dependence and assuming uniform angular emission.

The fraction of radiation intercepted by the detector was calculated as the ratio of its area (1 mm^2^) to the area of the hemisphere with the radius equal to the distance between the IR sensor and the IR source. This gave us the black-body radiation incident on the sensor area from the sample area of the black body. The values obtained for the Peltier device at different temperatures (28, 31, 34 and 37 °C) were then scaled by the ratio of the measured signals in mV (from the lockin amplifier) to the black-body signal. This adjustment enabled us to derive the intensity of the IR signal at different temperatures (28–37 °C).

Similarly, to calculate the IR signal intensity at different distances (8, 10, 15, 20, 25 and 30 cm) from the Peltier at 34 °C, the fraction of radiation intercepted by the detector was calculated as the ratio of its area (1 mm^2^) to the area of the hemisphere with the radius equal to the different distances between the IR sensor and the IR source. This gave us the black-body radiation incident on the sensor area from the sample area of the black body. The values obtained for the Peltier device at different distances (8–30 cm) were then scaled by the ratio of the measured signals in mV to the black-body signal. This adjustment enabled us to derive the intensity of the IR signal at different distances.

### Set-up to assay responses to IR blocking using IR filter window

We used silicon (Si) IR windows (100 mm diameter and 0.5 mm thickness with a small notch on the edge) that have a transmission range spanning 1.2–7 μm, although, even in this range, the Si wafer reduced the IR transmission. The Si wafer effectively blocked most of the IR wavelengths above 7 μm (Soka Technology, P100). As the blocking efficiency depends on the window thickness, we used multiple Si wafers to create a dose–response for IR blockage. Variations in thickness (1 mm, 2 mm and 3 mm) were used to effectively block IR from 34 °C IR source. The efficiency of the IR blockage was checked using IR thermography. As the dimensions of the Si wafer were different from the Peltier dimensions, we cut out cardboard to match the dimensions of the Si wafer and placed the cardboard with Si wafer in front of the 34 °C Peltier. The background of the images was normalized by setting the same colour space in the colour distribution settings of FLIR Ignite software. The IR-blocking experiments revealed that the 3 mm thick IR block window substantially blocked the IR from the 34 °C source. To perform the behavioural experiments, the same IR-blocking Si wafers were placed in front of both Peltiers to prevent any visual bias. Preference assays were conducted with wild-type (LVP) females in this modified set-up with different thicknesses of IR block windows, where one Peltier was at 34 °C and other at ambient 29.5 °C. Both sides were exposed to human odour and 5% CO_2_.

### Development of object-tracking MATLAB scripts

We used an automated tracking and scoring program for several reasons. First, automated video analysis substantially increases the throughput of experiments compared with other conventional manual scoring methods. Second, automated scoring reduces the opportunity for scoring bias that could arise during manual counting methods. Third, automated scoring enabled us to selectively study and score those mosquitoes actively host seeking on the cage mesh (Supplementary Video 1) and not those that are stationary.

We initially tried commercially available or open-source tracking programs; however, they were either too cumbersome, time consuming or ineffective in tracking mosquitoes well. We therefore developed a bespoke set of scripts to track and score our experiment recordings using MATLAB (MathWorks). All of the code described in this study is available at the Craig Montell Lab GitHub repository (https://github.com/Craig-Montell-Lab/Chandel_DeBeaubien_2023).

The tracking program generates a thresholded image (black and white) in which the mosquitoes appear as black blobs against a white background (Supplementary Fig. 1b,c). The centroid (centre) coordinates of these blobs are recorded and stored for every video frame of the experiment (99.8% were 5 min × 10 fps = 3,000 total frames). To selectively study host-seeking mosquitoes, we reconstructed the movements of the same mosquito over time. We used a nearest-neighbour function with a maximum-cut-off value to stitch together coordinates in successive frames. This method enabled us to reconstitute the trajectories of individual mosquitoes throughout the 5 min experiment recording (Supplementary Fig. 1d,e).

For the videos related to the effective distance for IR detection, we used a nearly identical approach to the one described above, only modifying our method for foreground detection. As there were small changes in the background of these videos as we changed the distance of the cage to the IR source, we opted to create a background model for each video rather than use a fixed pixel threshold. To accomplish this, we randomly extracted 100 frames from each video and then calculated the modal pixel value. The resulting image was used as the background model, as it was devoid of all moving objects (mosquitoes). To identify mosquitoes, we took the absolute difference in pixel value between each frame and the background model, thresholding pixel changes >30 arbitrary units. This robustly identified mosquito blobs in the foreground, which were then subjected to identical filtering as described above and below.

We analysed the mosquito movements to identify quantitative features that would enable us to study host-seeking mosquitoes selectively. First, we isolated the position data from mosquitoes that landed on the mesh of the cage, and removed data from mosquitoes that were flying. To do so, we analysed all of the blob areas (pixels) captured throughout a 5 min experimental recording. By analysing the distribution of body sizes, we found that those of landed mosquitoes fell within a defined range and were almost always larger than those of flying mosquitoes (Supplementary Fig. 1f). In video recordings, flying mosquitoes appeared less opaque than landed ones, causing their size to appear smaller after image thresholding. Thus, to selectively study mosquitoes that had landed, we thresholded the data of body sizes that fell within a defined range (Supplementary Fig. 1f).

Having isolated mosquitoes that landed on the cage mesh, we wanted to then isolate the data from mosquitoes that are actively host seeking. Here we define host seeking as walking along the cage mesh, which is correlated with probing behaviour (Supplementary Video 1). As we cannot directly observe probing from the vantage point of the recording camera, we use walking movement as a proxy for this behaviour. To empirically determine stationary and host-seeking behaviour features, we manually generated two representative datasets of stationary and seeking mosquito movements. These data were curated from actual experimental data. By analysing the distributions of velocities in these data, we determined a threshold value that, when exceeded, represents mosquitoes in motion (Supplementary Fig. 1g). Note that the seemingly paradoxical velocity of stationary mosquitoes results from slight differences in the calculated centroid position of that stationary object over time, called jitter. The remaining data after these steps represent actively host-seeking mosquitoes and were used for analysis in all behaviour experiments described in this study. The overall host seeking is referred to as the HSI, and is calculated as the total number of host-seeking observations during each video (99.8% were for 5 min), divided by the total number of frames (for example, 3,000 frames for the 5 min videos). In other words, the HSI represents the average number of mosquitoes that are host seeking at any given time. Moreover, for the experiments shown in Fig. 3a, we calculate the IHSI by dividing the total number of host-seeking mosquitoes in one zone at a given timepoint by the total number of experiments (18 experiments, 6 biological replicates, 3 technical replicates each). This metric represents the average number of mosquitoes host seeking in that zone, at that timepoint. As *An. stephensi* rarely walked around the back of the cage, we opted not to threshold the IR data based on walking speed and, instead, included data from all landing events.

The automatically determined HSI as described above does not merely measure walking but includes a requirement for probing with the proboscis. This is unlike previously used manual counting approaches that cannot discriminate between random landings and mosquitoes that display behaviour associated with blood seeking. We therefore compared the sensitivities of using the HSI with manually quantifying the number of mosquitoes in each zone every 30 s from the videos. We found that manual counting under-represents the preference for the IR zone in comparison to our automated scoring method (Extended Data Fig. 2c). In total, we recorded 1,483 videos (see Data availability section), 1,480 of which were for 5 minutes. The only exceptions were videos IR 1878 (3.60 min) and IR 1882 (4.89 min), corresponding to Fig. 3e, and SI 10 (4.82 min), corresponding to Fig. 3b.

### Optimization of scoring parameters

In this study, we use the PI metric to summarize mosquitoes’ biased or unbiased distribution during a given behaviour assay. This metric takes into account all observations of mosquito host seeking. Thus, if too few data points are fed into this metric, random variation significantly influences the experimental outcome. We wanted to determine an informed rationale for the minimum acceptable HSI for experiments to be included or excluded in this study. To investigate how low response rates impact the overall variance in experimental outcomes, we created a model informed by actual experiment parameters. We first analysed experimental data to identify key features of mosquito movements while host seeking. By examining the directionality of movements, we found that seeking mosquitoes walk mostly upwards with no left/right bias (Supplementary Fig. 1h). Using this information and their average velocities, we created a random walk simulation that approximates the movement duration, velocity and directionality of host-seeking mosquitoes (Supplementary Fig. 1i,j).

To model the effect of HSI on experiment variance, we populated a set number of fictive mosquitoes in a two-dimensional environment with the same dimensions as real experiments (720 × 1,280 total, with two 466 × 456 scoring zones). We simulated mosquito movement using the previously described movement model. The input number of mosquitoes ranged from 1 to 30 and, at each input number, the model was iterated 10,000 times. We then analysed the resulting movements in the same manner as for the real experimental data, recording both the PI and HSI. As the starting position was uniformly random, we would expect the average of simulated PI outcomes to approximate 0. We found that the distribution of outcomes with low HSIs had a very wide distribution and, in extreme cases, ranged from a PI of −0.99 to 0.95 (Extended Data Fig. 2d). These indicate that, at extremely low HSIs, the ability of the data to represent the underlying preference is poor. Furthermore, there was a nonlinear reduction in variance as the HSI increased (Extended Data Fig. 2d). With this information, we determined a minimum HSI of 5 to be required for the inclusion of an experiment in subsequent analyses. We chose this threshold because there is little additional decrease of variance at higher response levels and, technically, this would require increasing the number of mosquitoes per assay to achieve such HSIs. In this study we analysed 522 biological replicates. Each biological replicate included ≥3 technical replicates. 8% of all technical replicates were excluded because they did not meet the HSI threshold (HSI = 5). This resulted in 85.2% of all biological replicates being calculated from the mean of ≥3 technical replicates (445 out of 522), and 14.8% being calculated from 2 technical replicates (77 out of 522).

### Correlation studies

To determine what behaviour or behaviours were strongly associated with changes in the PI, we analysed 982 individual behaviour experiments. We first investigated whether a shift in PI was correlated with mosquitoes spending longer time on average in the preferred zone. To do this, we calculated the difference in ATT for each zone by dividing the cumulative host-seeking time spent in each zone by the overall number of tracks (bouts) observed in that zone (Extended Data Fig. 5a). The data for each experiment were normalized to the average host-seeking time spent in all zones, providing a metric with a range of −1 to 1. An ATT score of <1 indicates that, in that experiment, individual mosquitoes spent on average more time occupying zone 1 while host seeking compared with zone 2. An ATT score of >1 would, therefore, show the inverse.

The next metric that we analysed was the difference in ATD between each zone (Extended Data Fig. 5b). One explanation for a strong PI is that mosquitoes are more prone to leave one zone versus another, which would be shown in a skewed ATD score. This was derived in a similar manner to the ATT score, wherein we calculated a normalized ATD differential between zones 1 and 2. An ATD score of <1 would suggest that mosquitoes on average walked longer bout lengths in zone 1 as compared to in zone 2, and the inverse is true if ATD > 1.

Finally, we wanted to score the DTT between each zone. This would reflect the total number of mosquitoes navigating to that zone and exhibiting host-seeking behaviour (Extended Data Fig. 5d). To do this, we calculated the normalized difference in total number of tracks, ranging from −1 to 1. A DTT score of <1 indicates that, in that given experiment, there was a greater number of overall host-seeking bouts in zone 1, and conversely more in zone 2 when DTT > 1.

### RT–PCR and qPCR

To detect expression of *trpA1* and opsin mRNAs in the antennae using reverse transcription PCR (RT–PCR) and qPCR, we isolated RNA from 200 antennae using TRIzol Reagent (Thermo Fisher Scientific). We prepared cDNA using SuperScript III Reverse Transcriptase (Thermo Fisher Scientific) with oligo(dT) primers. For RT–PCR, we amplified for 32 cycles using Phusion High-Fidelity DNA Polymerase (New England BioLabs) and for qPCR, we amplified for 40 cycles using the LightCycler 480 SYBR Green I Master Mix (Roche). We used *RpL17* as the normalization reference. The PCR primers are listed below. The RNAs prepared from the control and mutants were used to produce the PCR products for the indicated genes (*trpA1*, *op1* and *op2*) and for *RpL17*. The PCR products for *trpA1*, *op1* and *op2* and for *RpL17* were loaded in adjacent sets of wells on the same 1% agarose gel. The original scan from two biological replicates is shown in Supplementary Fig. 3. The data shown in Extended Data Fig. 7a are from the experiment presented at the top in Supplementary Fig. 3.

The PCR primers were as follows: Op1-f (5568060), 5′-AGAAGAGAAATAGAATGGCGGC-3′; Op1-r (5568060), 5′-GAACGCTAGGTTGACCACCA-3′; Op2-f (5567680), 5′-CTGTCCGGAGGAGAAGACAATG-3′; Op2-r (5567680), 5′-GGTCAGGTAGTCAGTTCCGC-3′; Op3-f (5568061), 5′-GGCACTCACTCCTGGGATTC-3′; Op3-r (5568061), 5′-TCGTGAGCAGATACAGCCTTAATA-3′; Op4-f (5566757), 5′-ATCTGACCGTGGTGGATAGA-3′; Op4-r (5566757), 5′-GAAGTCCGAGAAGGCTAGATTG-3′; Op5-f (5566755), 5′-AACATGAGCGCTTGTGGAAC-3′; Op5-r (5566755), 5′-GTAACCGTTTCAACATTATCAAGTG-3′; Op7-f (5569125), 5′-CGTGGTCGCTGGGATTATT-3′; Op7-r (5569125), 5′-GATGGTGAACAGTGGGATGT-3′; Op8-f (5572198), 5′-ACTATCTGGCATTGGTGCTGG-3′; Op8-r (5572198), 5′-ATTTGCATGCACAAGCTGGG-3′; Op9-f (5576882), 5′-TTGCGACGGTGTTCTTTTGG-3′; Op9-r (5576882), 5′-CATTGCGTAAGGATTTTGATGTTGA-3′; Op10-f (5566350), 5′-CGCTACCGGGAATGTTTGGTG-3′; Op10-r (5566350), 5′-TCTTAGCAAGGATTGCGGGG-3′; Op12-f (5566410), 5′-GGCCAACATCAGTTGTTCCG-3′; Op12-r (5566410), 5′-TCGGCTATCGATTGGTTCCG-3′; trpA1-f (5571938), 5′-TGTTATCAAAGGTCTCAAGGATGA-3′; trpA1-r (5571938), 5′-AACAGGATTGGCATCAGTATCA-3′; RpL17-f (5574866), 5′-AAGAAGTGGCCATCATTCCA-3′; RpL17-r (5574866), 5′-GGTCTCCGGGTCGACTTC-3′; Op1-RT-f (5568060), 5′-CAACCTAGCGTTCTCGGATTT-3′; Op1-RT-r (5568060), 5′-GGCCCTTCACGATGACATTAT-3′; Op2-RT-f (5567680), 5′-CCAACCTGCTAGTGGTCAAT-3′; Op2-RT-r (5567680), 5′-GTAGACGAAGATGGCGTAGATG-3′; trpA1-RT-f (5571938), 5′-ACTGTAAACCGTCCATCGTTAG-3′; trpA1-RT-r (5571938), 5′-TATCTTGAGCGGTGTGGTAATC-3′.

### In situ hybridization

We devised a modified RNAscope^53^ protocol for whole-mount *Aedes* staining. In brief, *Ae. aegypti* probes were designed by Advanced Cell Diagnostics (ACD) to target genes Aae-LOC5571938 (2326–3239 bp of XM_021842755.1, *trpA1*), Aae-AAEL005776 (875–1770 bp of NM_001358471.1, *orco*), Aae-LOC5567680 (900–1397 bp of XM_001657569.3, *op*2), Aae-LOC5568060 (2–1378 bp of XM_001651947.3, *op1*), Aae-AAEL018153 (1202–2119 bp of XM_021844485.1, *brp*). RNAscope experiments were performed on whole-mount antennae in Eppendorf tubes. Around 10 antennae were dissected into PBS, washed once with PBS and fixed in 4% paraformaldehyde in PBS for 16 h at 4 °C. Each sample was then dehydrated in a series of 50% ethanol in PBS, 75% ethanol in PBS and 100% ethanol. After the last wash, ethanol was removed completely, and the tissues were air dried at room temperature for 30 min. The tissues were then treated with 3% H_2_O_2_ in PBS for 10 min to inactivate endogenous peroxidase activity. The samples were then incubated in RNAscope Protease III for 30 min. Probe hybridizations were performed overnight at 40 °C. The next day, the tissues were washed three times in RNAscope wash buffer (ACD, 310091) for 2 min each. The tissues were then incubated with amplifier solutions (Amp1–3) contained in the RNAscope Multiplex fluorescent V2 assay kit (ACD, 323100) according to the manufacturer’s instructions. The tissues were incubated in Amp1 for 2 h at 40 °C, in Amp2 for 2 h at 40 °C, Amp3 for 1 h at 40 °C and C1 for 2 h at 40 °C. Between each step, the tissues were washed five times for 3 min each at room temperature. For fluorescence labelling, a working Opal dye solution was made fresh using a 1:500 ratio of Opal dye (Akoya Biosciences) to TSA buffer. We then added 150 µl of the working solution to each tube containing around 10 antennae and incubated the samples at 40 °C for 2 h. The tissues were then washed in a wash buffer and mounted in VECTASHIELD mounting medium (Vector Laboratories). Experiments were repeated three times on control and mutant antennae samples. Images were acquired using the Zeiss LSM 900 confocal microscope and Leica SP8 resonant scanning confocal microscope. Maximum-intensity projections of full *z*-stacks were generated using ImageJ. Three-dimensional neuron counting in the acquired *z* stack of the 13th flagellomere was performed using Imaris (v.10.0.1). Each count was analysed manually in Imaris to remove objects that were not neurons and to add missing or overlapping neurons.

### Histology

Isolated antennae were primary fixed by placing them in a 0.1 M phosphate buffer (pH 7.2), 2% glutaraldehyde solution overnight at 4 °C, and then secondary fixed in 0.1 M phosphate buffer (pH 7.2), 1% osmium tetroxide solution for >2 h. The fixed antennal samples were then dehydrated with serial dilutions of ethanol and acetone, infiltrated with epoxy resin (Electron Microscopy Sciences, 14310), embedded in PE moulding trays and cured in an oven. The cured preparations were sectioned at a thickness of approximately 1 µm using a glass knife on a Reichert-Jung Ultracut microtome, stained with toluidine blue and observed under a light microscope.

### EAG recordings

We used 5–10-day-old female mosquitoes to perform EAG recordings. Mosquitoes were immobilized on glass slides by attaching their thoraxes and abdomens to strips of double-sided adhesive tape. The mosquito heads rested on top of a coverslip pre-attached to the slide. Thin strips of double-sided transparent tape were used to secure the proboscis to minimize movement. The antennae used for the recordings were immobilized on coverslips with a thin strip of double-sided transparent tape placed along the middle part of the antennae. Glass electrodes (World Precision Instruments, 1B150F-3) were pulled on a Sutter Instrument P97 puller and filled with Beadle–Ephrussi Ringer solution. The electrodes were inserted into drops of electrode cream (Parker Laboratories Cream Electrode Signacreme Ea, 72 BT/CA; 17-05) placed on the compound eye (reference electrode) and near the proximal end of the 13th flagellomere of the antenna (recording electrode).

To deliver the IR stimuli, water from a hot water bath maintained at 37 °C was circulated through an aluminium plate (4 cm × 4 cm × 1 cm) so that the final temperature of the surface was 34 °C. The stimulus was applied by placing the heated plate 3 cm from the antenna using a manipulator, with the broader 4 cm square surface of the plate facing the antenna. This surface was covered with white ConTact paper (emissivity, 0.92). To establish that the source of IR (the 34 °C block) did not change the temperature at the site of the preparation due to conductive heat, we measured the ambient temperature.

To test whether the mosquitoes were responsive to stimuli, we exposed them to a positive control (mouth puff) before exposure to the 34 °C stimuli, and determined whether or not there was a response in the trace. If a mosquito did not elicit an EAG response to the positive control, it was excluded from the analysis. If there was no response to the IR, we performed a positive control after the stimulus. If the response to the mouth puff was negative, we excluded the data. To measure the change in field potential after stimulus application, the EAG signals were amplified using the IDAC-4 amplifier and digitized using the EAGpro software (Ockenfels SYNTECH). The following formula was used to measure the amplitude change following each stimulus: (value of the peak response within 5 s after stimulus) − (average field potential for 5 s before the stimulus).

### Statistical methods

Data from preliminary experiments were used to determine the typical s.d. in behavioural experiment results (*σ*_*x*_ = 0.12). We predetermined an effect size of ±0.2 change in the PI to be of interest, and therefore an *n* of 6 replicates for each treatment would be sufficiently powered. All behavioural experiment groups (such as genotype, condition) consisted of 6 (*n* = 6) biological cohorts (biological replicates), and repeated measurements (technical replicates) were averaged. For experiments designed with two groups, significant differences in group means were determined using parametric two-tailed Student’s *t*-tests. For experiments with >2 treatment groups, differences in group means were analysed using one-way ANOVA followed by a Tukey’s multiple-comparison test. Significance is indicated by asterisks.

### Reporting summary

Further information on research design is available in the Nature Portfolio Reporting Summary linked to this article.

## Online content

Any methods, additional references, Nature Portfolio reporting summaries, source data, extended data, supplementary information, acknowledgements, peer review information; details of author contributions and competing interests; and statements of data and code availability are available at 10.1038/s41586-024-07848-5.

## Supplementary information

Supplementary Information Supplementary Discussion, Supplementary Figs. 1–3, Supplementary References and the legends for Supplementary Videos 1 and 2.

Reporting Summary

Supplementary Video 1 Simultaneously recording probing and walking behaviours. Probing behaviour is correlated with walking behaviour. As the females are actively host seeking, they extend their proboscis through the cage mesh in an effort to make contact with a host-like surface. When they fail to make contact, they withdraw their proboscis and continue walking along the cage mesh. The walking track is shown in magenta with dots indicating recorded probing events. Left, walking behaviour observed from inside the cage. Right, probing behaviour recorded from outside the cage.

Supplementary Video 2 Representative IR preference assay. The positions of host-seeking female mosquitoes (magenta) are recorded throughout the 5 min experimental window. The overall distribution of recorded positions was used to calculate the preference index. Left zone, 5% CO_2_ and human odour. Right zone, 5% CO_2_, human odour and IR from a 34 °C Peltier device.

## Source data

Source Data Fig. 2

Source Data Fig. 3

Source Data Fig. 5

Source Data Extended Data Fig. 1

Source Data Extended Data Fig. 2

Source Data Extended Data Fig. 3

Source Data Extended Data Fig. 4

Source Data Extended Data Fig. 5

Source Data Extended Data Fig. 6

Source Data Extended Data Fig. 7

Source Data Extended Data Fig. 8

## Data Availability

All raw video data have been uploaded to Dryad^54^ (10.5061/dryad.547d7wmh3). Source data are provided with this paper.

## Code availability

The code/scripts generated in this study are available at GitHub (https://github.com/Craig-Montell-Lab/Chandel_DeBeaubien_2023).
