# Supplementary material for: Thermal infrared directs host-seeking behaviour in Aedes aegypti mosquitoes
Source: Nature. 2024 Aug 21;633(8030):615–23. doi: 10.1038/s41586-024-07848-5 (PMC11410652; doi:10.1038/s41586-024-07848-5)
Supplement: Supplementary file 1 — Supplementary Discussion, Supplementary Figs. 1–3, Supplementary References and the legends for Supplementary Videos 1 and 2. [file 41586_2024_7848_MOESM1_ESM.pdf]

nature portfolio

https://doi.org/10.1038/s41586-024-07848-5

Supplementary information

Thermal infrared directs host-seeking behaviour in *Aedes aegypti* mosquitoes

In the format provided by the authors and unedited

Supplementary Information

Thermal infrared directs host-seeking behavior in *Aedes aegypti* mosquitoes

Avinash Chandel[1,2*], Nicolas A. DeBeaubien[1,2*], Anindya Ganguly[1,2], Geoff T. Meyerhof[1,2], Andreas A. Krumholz[3,4], Jiangqu Liu[1,2], Vincent L. Salgado[3,5], and Craig Montell[1,2,**]

[1]Department of Molecular, Cellular, and Developmental Biology, University of California, Santa Barbara, CA, USA

[2]Neuroscience Research Institute, University of California, Santa Barbara, CA, USA

[3]BASF Corp, Research Triangle Park, NC, USA

[4]Südzucker AG, Mannheim, Germany

[5]Duke University Department of Biology, Durham, NC, USA

[*]These authors contributed equally to this work.

[**]Corresponding author. Email: cmontell@ucsb.edu

Supplementary Discussion

Our work reveals that thermal IR is an effective mid-range cue used by *Ae. aegypti* in combination with other host-derived cues such as CO_2 and organic human-derived odors. This finding expands our understanding of the repertoire of host-derived cues employed by blood-sucking mosquitoes to zero in on their hosts. In addition to sensing IR, CO_2 and organic odors at a distance, once the female mosquitoes are <10 cm from their host, additional cues come into play. These include convective heat[3,9,14,16,17,28], humidity[18] and chemicals with low to moderate volatility that can only be detected within close proximity. After the mosquitoes land on their host, they sense conductive heat from the body surface[16], and sample many gustatory cues on the skin, which contribute to the mosquitoes' decision to probe for a blood meal, or fly away[55].

While *Aedes* are capable of sensing many host cues, they cannot sense all of them accurately under all environmental conditions[8]. *Ae. aegypti* are most active during the day, and seek out a silhouette that they interpret as a potential human. However, in a completely dark environment, vision is eliminated. The effectiveness of human odors as accurate directional cues is decreased if the host is moving quickly, or if there is strong wind[8]. The efficacy of thermal IR is also affected by the environment. When the environmental temperature matches that of human skin, thermal IR is not a useful attractant. Moreover, some clothing reduces thermal IR since the emissivity of clothing varies depending on the material, and how loose-fitting the clothing is to the skin[56,57] (**Supplementary Fig. 2**).

Since there are times when certain host-associated cues cannot be sensed and used optimally, the larger the repertoire of stimuli that mosquitoes have at their disposal to locate preferred targets, the more likely they will be successful in obtaining a blood meal. The detection of thermal IR is an important part of the sensory arsenal that mosquitoes employ to find hosts since its effectiveness is not compromised by the same environmental conditions that interfere with vision (darkness), and CO_2 as well as organic odorants (strong wind and rapid movement of the host)[8].

Several lines of evidence support our conclusion that neurons in the peg-in-pit coeloconic sensilla at the distal end of the antenna house the thermosensory neurons that detect thermal IR. These include the demonstration that thermal IR detection is eliminated if the distal part of the antenna is removed, and that two opsins (Op1 and Op1) and the TRPA1 channels contribute to IR detection, and are co-expressed in neurons near the antennal tip. The thermosensitive sensilla of blood-feeding triatomine ('kissing') bugs are also coeloconic[58]. The peg-in-pit structure of these sensilla is important for their function as directional thermal IR sensors, since their shape limits the angle from which its temperature-sensing dendrites receive radiation. In fact, all previously identified IR-sensing organs used for sensing warm-blooded prey are pit-type organs. The pit organs of pit vipers are comprised of a thin membrane, which is densely innervated by thermosensitive neurons, and suspended for thermal isolation within a hollow pit that serves to limit the field of view[21,59]. The IR-sensing anterior capsule of the tick Haller's organ also has a peg-shaped sensilla in a pit[27].

Our findings that two opsins and TRPA1 function in detection of thermal IR are reminiscent of the contributions of several *Drosophila* opsins and *Drosophila* TRPA1 for thermotransduction and for tasting aversive bitter compounds such as aristolochic acid[45,46,60]. *Drosophila* TRPA1 is required for avoiding warm temperatures above the thermal threshold for activation of the channel[38,39,61-63], and for repulsion to high levels of aristolochic acid that directly activate TRPA1[60]. However, lower temperatures and more dilute concentrations of aristolochic acid that are insufficient to directly activate TRPA1, still require TRPA1 for avoidance[45,46,60]. In these latter cases, the lower temperatures and levels of aristolochic acid activate opsins, which initiate signaling cascades that result in indirect activation of *Drosophila* TRPA1.

Pit viper snakes, which detect thermal IR from prey through their pit organs, do so through a warm-activated TRPA1 channel expressed in neurons innervating this organ[21]. *Ae. aegypti* diverged from pit viper snakes such as *Crotalus atrox* (western diamondback rattlesnake) ~700,000,000 years ago (timetree.org). Thus, a role for TRPA1 in detecting radiant heat appears to be ancient, although we cannot rule out that this represents a convergent function. It has been ~250,000,000 years since *Drosophila* and *Aedes* shared a common ancestor (timetree.org). Since opsins function in thermotransduction in *Drosophila*[45,46], the finding that *Aedes* Op1 and Op2 contribute to sensing thermal IR in *Aedes* illustrates the ancient roles for opsins for thermotransduction. It is intriguing to speculate that one or more opsins may also be expressed in pit organs and enable viper snakes to detect radiant heat from prey at a greater distance than is sufficient to directly activate snake TRPA1.

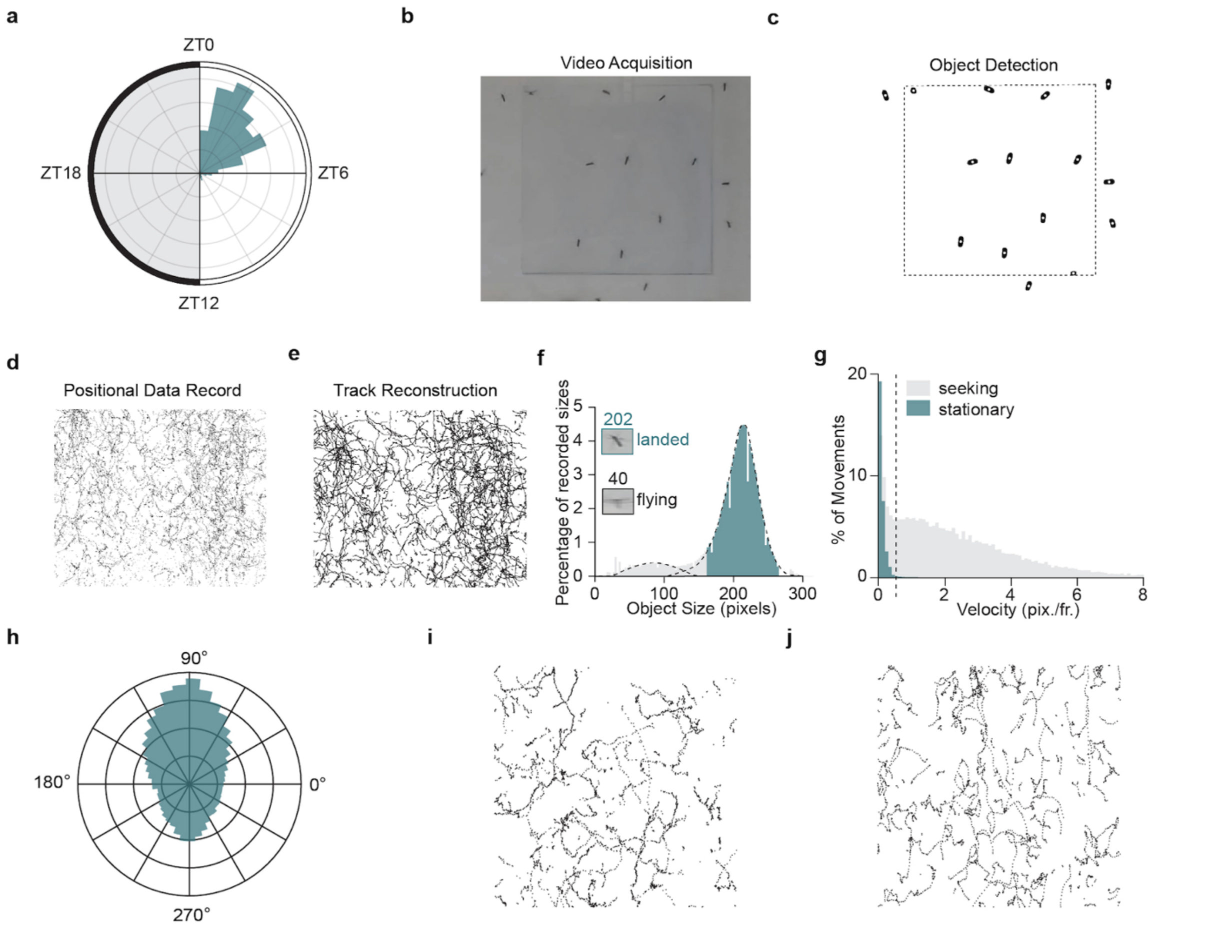

Supplementary Fig. 1 I Data acquisition during video recordings of behavior. All data were collected from females exposed to 5% CO_2, human odor and thermal IR from a Peltier device at 34°C. **a,** Histogram of experimental start times. The majority of behavioral experiments were restricted to the mosquito subjective morning (ZT1 – ZT5). **b,** Sample frame from a video during an IR behavioral assay. **c,** Results of image segmentation and mosquito object detection. **d,** Depiction of positional data recorded throughout the five-minute experiment window. **e,** Walking tracks of mosquitoes reconstructed from positional data. **f,** Distribution of recorded object sizes from the experimental data reveals a bimodal distribution of landed and flying mosquitoes. Shown in the insets are representative images of landed and flying mosquitoes and their observed object sizes. The data from flying mosquitoes were not used for subsequent analysis. The subsequent analyses used the landed mosquitoes within the indicated boundaries (green). The dotted lines are added for emphasis. The object size threshold used to isolate landed mosquitoes is indicated in the shaded region. **g,** Histogram depiction of manually curated walking (gray) and stationary (green) mosquitoes. A minimum velocity threshold (dashed line) was used to selectively score walking, host-seeking mosquitoes. pix., pixels; fr., frame. **h,** Directionality of mosquito walking/probing from the behavioral data. **i,** Representative mosquito walking traces. **j,** Representative fictive walking traces used in the behavior model.

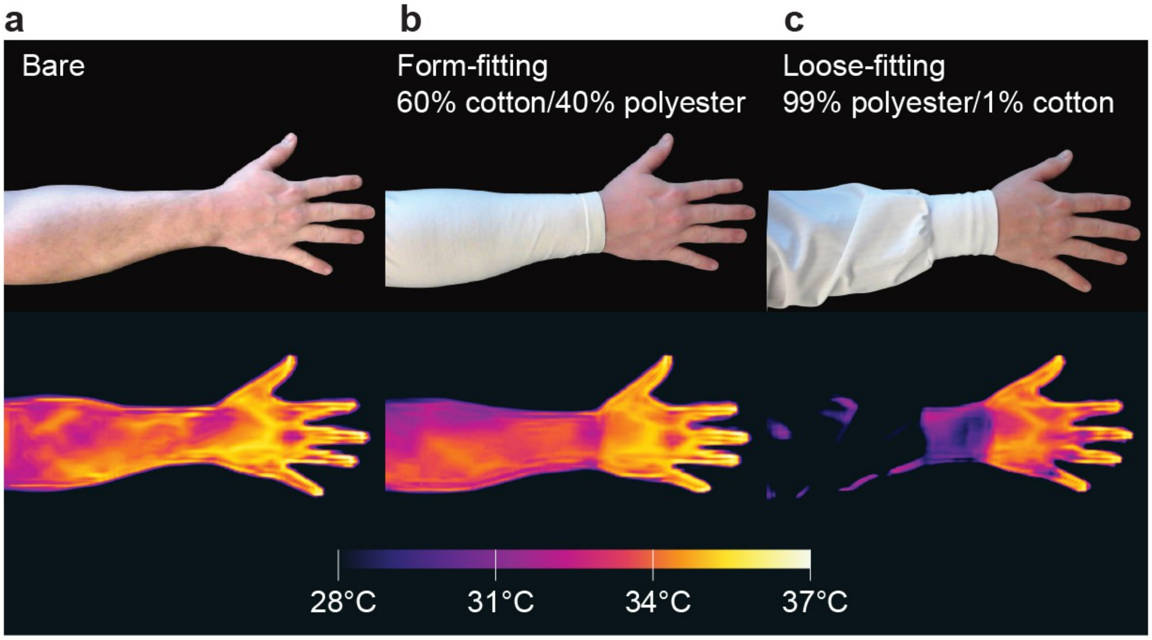

Supplementary Fig. 2 I Examples of the effects of clothing on IR emissivity. The same human arm and hand were imaged with a FLIR One infrared smartphone camera under three conditions. **a,** Bare arm and hand. **b,** Arm covered with form-fitting clothing (60% cotton and 40% polyester). **c,** Arm covered with loose-fitting clothing (99% polyester and 1% cotton). Note that the thermal emission increased when the same material fit tightly around the wrist.

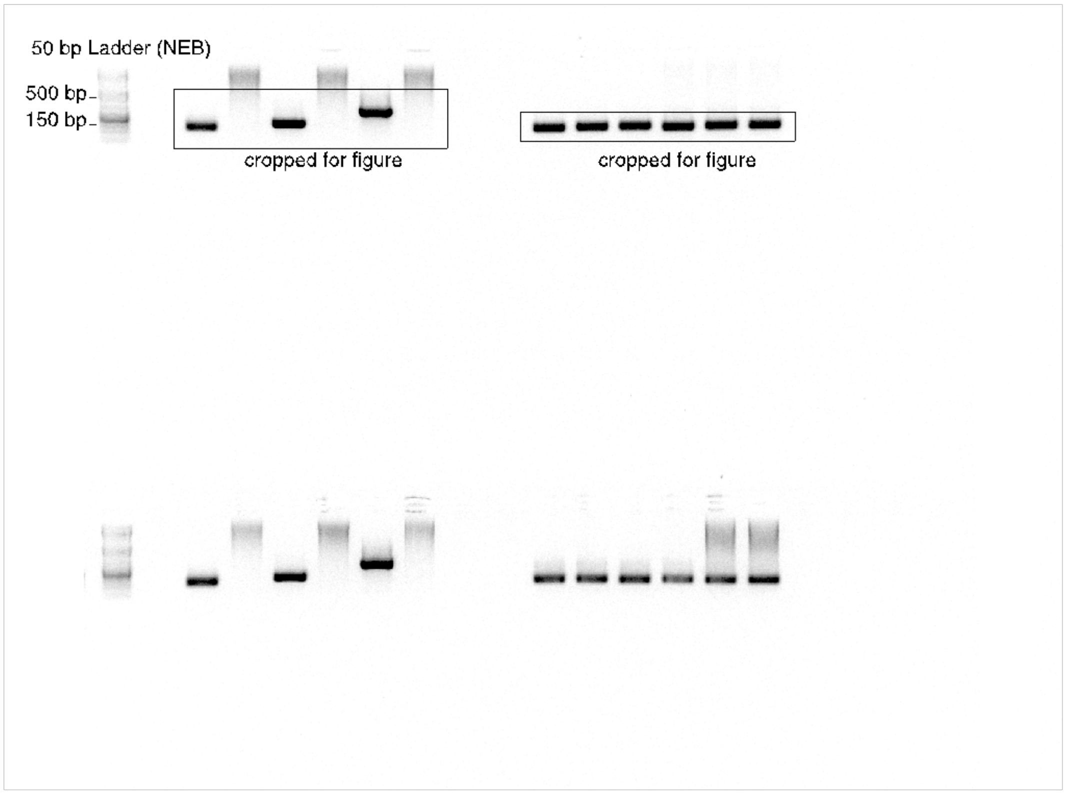

Supplementary Fig. 3 | Uncropped RT-PCR gel shown in **Extended Data Figure 7a**.

Supplementary Discussion References

55 Sparks, J. T. & Dickens, J. C. Mini review: Gustatory reception of chemicals affecting host feeding in aedine mosquitoes. *Pestic. Biochem. Physiol.* **142**, 15-20 (2017).

56 Yuce, I. *et al.* Titanium and Silicon Dioxide-Coated Fabrics for Management and Tuning of Infrared Radiation. *Sensors* **22** (2022).

57 Zhang, H., Hu, T. & Zhang, J. Transmittance of infrared radiation through fabric in the range 8–14 µm. *Text. Res. J.* **80**, 1516-1521 (2010).

58 Zopf, L. M., Lazzari, C. R. & Tichy, H. Differential effects of ambient temperature on warm cell responses to infrared radiation in the bloodsucking bug Rhodnius prolixus. *J. Neurophysiol.* **111**, 1341-1349 (2014).

59 Campbell, A. L., Naik, R. R., Sowards, L. & Stone, M. O. Biological infrared imaging and sensing. *Micron* **33**, 211-225 (2002).

60 Leung, N. Y. *et al.* Function of opsins in *Drosophila* taste. *Curr. Biol.* **30**, 1367-1379 (2020).

61 Rosenzweig, M., Kang, K. & Garrity, P. A. Distinct TRP channels are required for warm and cool avoidance in *Drosophila melanogaster*. *Proc. Natl. Acad. Sci. U.S.A.* **105**, 14668-14673 (2008).

62 Zhong, L. *et al.* Thermosensory and non-thermosensory isoforms of *Drosophila melanogaster* TRPA1 reveal heat sensor domains of a thermoTRP channel. *Cell Rep.* **1**, 43-55 (2012).

63 Kang, K. *et al.* Modulation of TRPA1 thermal sensitivity enables sensory discrimination in *Drosophila*. *Nature* **481**, 76-80 (2012).
