# Supplementary material for: Thermal infrared directs host-seeking behaviour in Aedes aegypti mosquitoes
Source: Nature. 2024 Aug 21;633(8030):615–23. doi: 10.1038/s41586-024-07848-5 (PMC11410652; doi:10.1038/s41586-024-07848-5)
Supplement: Supplementary file 2 — Reporting Summary [file 41586_2024_7848_MOESM2_ESM.pdf]

nature portfolio

Corresponding author(s): Craig Montell

Last updated by author(s): Jun 1, 2024

Reporting Summary

Nature Portfolio wishes to improve the reproducibility of the work that we publish. This form provides structure for consistency and transparency in reporting. For further information on Nature Portfolio policies, see our Editorial Policies and the Editorial Policy Checklist.

Statistics

For all statistical analyses, confirm that the following items are present in the figure legend, table legend, main text, or Methods section.

n/a	Confirmed	
☐	☒	The exact sample size (*n*) for each experimental group/condition, given as a discrete number and unit of measurement
☐	☒	A statement on whether measurements were taken from distinct samples or whether the same sample was measured repeatedly
☐	☒	The statistical test(s) used AND whether they are one- or two-sided<br>*Only common tests should be described solely by name; describe more complex techniques in the Methods section.*
☒	☐	A description of all covariates tested
☒	☐	A description of any assumptions or corrections, such as tests of normality and adjustment for multiple comparisons
☐	☒	A full description of the statistical parameters including central tendency (e.g. means) or other basic estimates (e.g. regression coefficient) AND variation (e.g. standard deviation) or associated estimates of uncertainty (e.g. confidence intervals)
☐	☒	For null hypothesis testing, the test statistic (e.g. *F*, *t*, *r*) with confidence intervals, effect sizes, degrees of freedom and *P* value noted<br>*Give P values as exact values whenever suitable.*
☒	☐	For Bayesian analysis, information on the choice of priors and Markov chain Monte Carlo settings
☒	☐	For hierarchical and complex designs, identification of the appropriate level for tests and full reporting of outcomes
☒	☐	Estimates of effect sizes (e.g. Cohen's *d*, Pearson's *r*), indicating how they were calculated

Our web collection on statistics for biologists contains articles on many of the points above.

Software and code

Policy information about availability of computer code

Data collection	Video Recording -- Behavior assays were video recorded using the Logitech Webcam Software (v2.51)<br>Electroantennogram -- EAG recordings were recorded using EAGPro software (Version dd: Nov 12 1018)<br>CO2 Measurements - Measurements recorded with GasLab (v.2.3.1.4)<br>Arduino Programming - Arduino controller programmed with Arduino IDE (v1.8.19)
Data analysis	Modeling -- The modeling described in Extended Data Fig. 6h,i and Extended Data Fig 3 were custom made in MATLAB (v9.6.0.1135713)<br>Video Analysis -- Behavior experiment videos were analyzed and scored using custom MATLAB scripts (v.9.6.0.1135713)<br><br>Source code can be found at: https://github.com/Craig-Montell-Lab/Chandel_DeBeaubien_2023

For manuscripts utilizing custom algorithms or software that are central to the research but not yet described in published literature, software must be made available to editors and reviewers. We strongly encourage code deposition in a community repository (e.g. GitHub). See the Nature Portfolio guidelines for submitting code & software for further information.

Data

Policy information about availability of data

All manuscripts must include a data availability statement. This statement should provide the following information, where applicable:

- Accession codes, unique identifiers, or web links for publicly available datasets
- A description of any restrictions on data availability
- For clinical datasets or third party data, please ensure that the statement adheres to our policy

All underlying data for graphical summaries are included in the Source Data.

Human research participants

Policy information about studies involving human research participants and Sex and Gender in Research.

Reporting on sex and gender	Humans research participants were not used in this study.
Population characteristics	Humans research participants were not used in this study.
Recruitment	Humans research participants were not used in this study.
Ethics oversight	Humans research participants were not used in this study.

Note that full information on the approval of the study protocol must also be provided in the manuscript.

Field-specific reporting

Please select the one below that is the best fit for your research. If you are not sure, read the appropriate sections before making your selection.

☒ Life sciences ☐ Behavioural & social sciences ☐ Ecological, evolutionary & environmental sciences

For a reference copy of the document with all sections, see nature.com/documents/nr-reporting-summary-flat.pdf

Life sciences study design

All studies must disclose on these points even when the disclosure is negative.

Sample size	Data from preliminary behavior experiments were used to determine the typical standard deviation for Preference Index (σx = 0.12). We predetermined an effect size of +/- 0.2 change in Preference Index to be of interest, and therefore an n of 6 replicates for each treatment would be sufficiently powered. We used an n of 6 for each treatment group in all behavior experiments in this study. For Fig. 1g these are representative data from an example experiment. For the EAG data ("Fig. 6a" in the original submission, now Fig. 5d) sample sizes were determined based on similar studies in the field.
Data exclusions	For behavioral data where preference was measured (PI), technical replicates with an HSI greater than or equal to 5 were included unless otherwise specified. A detailed description of this threshold is described in "Optimization of Scoring Parameters." If a given treatment group failed to achieve an HSI greater or equal to 5 in at least two technical replicates, it was excluded from reporting.
Replication	Each genotype or condition was assayed with n = 6 biological replicates, each assayed a minimum of 3 technical replicates, then averaged. See "Data Exclusions" for a detailed explanation of replicate exclusion. The Source Data includes all underlying replicates for behavioral experiments. All attempts at replication were successful.
Randomization	Female mosquitoes were randomly aspirated from mixed-sex reading cages and placed in assay cages prior to experimentation.
Blinding	Investigators were not blinded to group allocation during data collection and analysis. This was not possible as typically only one experimental variable was assayed in a given day (ex. genotype, temperature, etc.) and therefore could not be sufficiently blinded by the researcher doing the mosquito rearing and/or behavior assays. Bias in assay scoring was mitigated through the use of automated video analysis and scoring scripts.

Reporting for specific materials, systems and methods

We require information from authors about some types of materials, experimental systems and methods used in many studies. Here, indicate whether each material, system or method listed is relevant to your study. If you are not sure if a list item applies to your research, read the appropriate section before selecting a response.

Materials & experimental systems

n/a	Involved in the study
☒	☐ Antibodies
☒	☐ Eukaryotic cell lines
☒	☐ Palaeontology and archaeology
☐	☒ Animals and other organisms
☒	☐ Clinical data
☒	☐ Dual use research of concern

Methods

n/a	Involved in the study
☒	☐ ChIP-seq
☒	☐ Flow cytometry
☒	☐ MRI-based neuroimaging

Animals and other research organisms

Policy information about studies involving animals; ARRIVE guidelines recommended for reporting animal research, and Sex and Gender in Research

Laboratory animals	Aedes aegypti strains: Liverpool (LVP), Orlando (ORL), trpA1[1], op1[1], op1[2], op2[1], op2[2], Gr19[1]; Anopheles stephensi. Mosquitoes were assayed/measured when 1-3 weeks old.
Wild animals	The study did not involve wild animals.
Reporting on sex	The findings of this study solely relate to female Aedes aegypti and Anopheles stephensi.
Field-collected samples	The study did not involve samples collected from the field.
Ethics oversight	The Arthropod Containment Level 2 (ACL-2) facility and the protocols for housing and characterizing Aedes aegypti were approved as outlined in the UCSB ACL2 Safety and Security Plan (BUA-21). This plan was also approved by the the Vector-Borne Disease Section of the California Department of Public Health (CDPH).

Note that full information on the approval of the study protocol must also be provided in the manuscript.
